# Correction: Psychometric properties and factor structure of the Early Development Instrument in a sample of Jordanian children

**DOI:** 10.1186/s40359-023-01462-2

**Published:** 2024-02-14

**Authors:** Emad G. Ababneh, Eric K. Duku, Caroline Reid-Westoby, Ashley Gaskin, Magdalena Janus

**Affiliations:** 1 National Center for Human Resources Development, Amman, Jordan; 2 Jadara University, Irbid, Jordan; 3 Amman Arab University, Amman, Jordan; 4 Department of Psychiatry and Behavioural Neurosciences, Offord Centre for Child Studies, McMaster University, 293 Wellington St, North, Suite 132, Hamilton, ON L8L 8E7 Canada

**BMC Psychology (2022) 10:316**

10.1186/s40359-022-01014-0

Following publication of the original article, it came to the authors’ attention that there were two typos in the syntax used for data analysis. After correcting the typos and redoing the analysis, they discovered that a number of corrections needed to be made to the article body and the additional file of the article. For details of these corrections, please see the two additional files 1 and 2 linked to this erratum.

The published article has been updated with these corrections. The authors thank you for reading this erratum and apologize for any inconvenience caused.

### Electronic supplementary material

Additional file 1. Corrected article body

Additional file 2. Corrected additional file

