# Supplementary material for: Psychometric properties and factor structure of the Early Development Instrument in a sample of Jordanian children
Source: BMC Psychol. 2022 Dec 21;10:316. doi: 10.1186/s40359-022-01014-0 (PMC9769466; doi:10.1186/s40359-022-01014-0)
Supplement: Supplementary file 1 — Additional file 1. Supplementary material. [file 40359_2022_1014_MOESM1_ESM.docx]

**6. Appendices**

**6.1. Appendix A**

**Table A1**

***P-values from student’s t-tests and effect sizes (Cohen’s d) of the differences between subgroups for demographic and contextual characteristics for the developmental domains of the EDI and, accounting for clustering within schools***

| Variables | Physical Health & Well-being | | Social Competence | | Emotional Maturity | | Language & Cognitive Development | | Communication Skills & General Knowledge | |
| --- | --- | --- | --- | --- | --- | --- | --- | --- | --- | --- |
|  | p-value | effect size | p-value | effect size | p-value | effect size | p-value | effect size | p-value | effect size |
| Child’s  sex | <0.001 | -0.158 | <0.001 | -0.248 | <0.001 | -0.400 | <0.001 | -0.137 | <0.001 | -0.183 |
| Child’s  age | 0.120 | -0.039 | <0.001 | -0.150 | 0.063 | -0.048 | <0.001 | -0.143 | <0.001 | -0.159 |
| Mother’s education | <0.001 | -0.305 | <0.001 | -0.408 | <0.001 | -0.190 | <0.001 | -0.488 | <0.001 | -0.458 |
| Father’s education | <0.001 | -0.241 | <0.001 | -0.390 | 0.001 | -0.138 | <0.001 | -0.457 | <0.001 | -0.428 |
| School location | 0.081 | -0.102 | <0.001 | -0.223 | 0.053 | -0.111 | 0.001 | -0.178 | <0.001 | -0.222 |

**6.2. Appendix B**

**Table B1**

***Results of the Confirmatory Factor Analysis: Goodness of fit statistics for the domains – 2010***

|  | χ^2^, df, p-value | RMSEA | CFI | TLI | WRMR |
| --- | --- | --- | --- | --- | --- |
| Physical Health & Well-being | 292.071, 62, <0.0001 | 0.030  (0.026, 0.033) | 0.982 | 0.977 | 1.567 |
| Social Competence | 5576.824, 293, <0.0001 | 0.065  (0.064, 0.067) | 0.952 | 0.947 | 3.761 |
| Emotional Maturity | 5226.729, 399, <0.0001 | 0.054  (0.052. 0.055) | 0.919 | 0.911 | 3.521 |
| Language and Cognitive Development | 1394.885, 293, <0.0001 | 0.031  (0.028, 0.031) | 0.972 | 0.969 | 2.045 |
| Communication Skills and General Knowledge | 642.514, 20, <0.0001 | 0.086  (0.080, 0.092) | 0.986 | 0.981 | 2.871 |

*Note.* RMSEA = Root mean square error of approximation, CFI = Comparative fit index, TLI = Tucker-Lewis index, WRMR = Weighted Root Mean Square Residual, χ^2^ = chi-square test of fit, df = degrees of freedom

**6.3. Appendix C**

**Table C1**

***Results of the Confirmatory Factor Analysis: Goodness of fit statistics for the five domains of the EDI – 2010***

|  | χ^2^, df, p-value | RMSEA | | CFI | TLI | WRMR |
| --- | --- | --- | --- | --- | --- | --- |
| Physical Health & Well-being | 380.800, 62, <0.0001 | 0.029  (0.027, 0.032) | 0.986 | | 0.983 | 1.693 |
| Social Competence | 7629.134, 293, <0.0001 | 0.065  (0.064, 0.066) | 0.946 | | 0.940 | 4.471 |
| Emotional Maturity | 7594.239, 399, <0.0001 | 0.055  (0.054. 0.056) | 0.916 | | 0.908 | 4.866 |
| Language and Cognitive Development | 1748.280, 293, <0.0001 | 0.029  (0.028, 0.030) | 0.975 | | 0.972 | 2.440 |
| Communication Skills and General Knowledge | 787.163, 20, <0.0001 | 0.080  (0.075, 0.085) | 0.988 | | 0.984 | 3.462 |

*Note.* RMSEA = Root mean square error of approximation, CFI = Comparative fit index, TLI = Tucker-Lewis index, WRMR = Weighted Root Mean Square Residual, χ^2^ = chi-square test of fit, df = degrees of freedom

**6.4. Appendix D**

**Table D1**

***Results of the Categorical Confirmatory Factor Analysis 2018 – Standardized factor loadings and thresholds for the EDI domains***

| Domains | Estimate | S.E. | Est./S.E. | Two-Tailed P-Value |
| --- | --- | --- | --- | --- |
| *Physical Health and Well-being* |  |  |  |  |
| F1 BY |  |  |  |  |
| PHWB01 | 0.762 | 0.032 | 23.634 | <0.001 |
| PHWB02 | 0.905 | 0.019 | 47.266 | <0.001 |
| PHWB03 | 0.870 | 0.018 | 47.213 | <0.001 |
| PHWB04 | 0.857 | 0.023 | 36.756 | <0.001 |
| F2 BY |  |  |  |  |
| PHWB05 | 0.725 | 0.039 | 18.412 | <0.001 |
| PHWB06 | 0.472 | 0.040 | 11.872 | <0.001 |
| PHWB07 | 0.936 | 0.032 | 29.343 | <0.001 |
| PHWB13 | 0.387 | 0.057 | 6.841 | <0.001 |
| F3 BY |  |  |  |  |
| PHWB08 | 0.911 | 0.009 | 102.954 | <0.001 |
| PHWB09 | 0.960 | 0.006 | 171.162 | <0.001 |
| PHWB10 | 0.857 | 0.014 | 61.241 | <0.001 |
| PHWB11 | 0.806 | 0.012 | 65.550 | <0.001 |
| PHWB12 | 0.836 | 0.015 | 55.940 | <0.001 |
| F2 WITH |  |  |  |  |
| F1 | 0.304 | 0.051 | 5.999 | <0.001 |
| F3 WITH |  |  |  |  |
| F1 | 0.412 | 0.035 | 11.864 | <0.001 |
| F2 | 0.590 | 0.034 | 17.498 | <0.001 |
| Thresholds |  |  |  |  |
| PHWB01$1 | -1.183 | 0.051 | -23.372 | <0.001 |
| PHWB02$1 | -1.110 | 0.038 | -29.583 | <0.001 |
| PHWB03$1 | -1.180 | 0.038 | -31.311 | <0.001 |
| PHWB04$1 | -1.213 | 0.045 | -26.846 | <0.001 |
| PHWB05$1 | -1.617 | 0.054 | -30.025 | <0.001 |
| PHWB06$1 | -0.716 | 0.051 | -14.017 | <0.001 |
| PHWB07$1 | -1.388 | 0.042 | -33.315 | <0.001 |
| PHWB08$1 | -2.299 | 0.054 | -42.281 | <0.001 |
| PHWB08$2 | -1.156 | 0.039 | -29.743 | <0.001 |
| PHWB09$1 | -2.224 | 0.053 | -41.914 | <0.001 |
| PHWB09$2 | -0.973 | 0.038 | -25.779 | <0.001 |
| PHWB10$1 | -2.303 | 0.059 | -38.719 | <0.001 |
| PHWB10$2 | -1.219 | 0.045 | -27.169 | <0.001 |
| PHWB11$1 | -1.951 | 0.050 | -39.064 | <0.001 |
| PHWB11$2 | -0.721 | 0.034 | -21.238 | <0.001 |
| PHWB12$1 | -2.299 | 0.065 | -35.382 | <0.001 |
| PHWB12$2 | -1.119 | 0.038 | -29.355 | <0.001 |
| PHWB13$1 | -1.789 | 0.074 | -24.129 | <0.001 |
| PHWB13$2 | -1.229 | 0.059 | -20.875 | <0.001 |
| *Social competence* |  |  |  |  |
| F1 BY |  |  |  |  |
| SOC01 | 0.851 | 0.013 | 65.507 | <0.001 |
| SOC02 | 0.870 | 0.010 | 84.910 | <0.001 |
| SOC03 | 0.909 | 0.009 | 106.589 | <0.001 |
| SOC04 | 0.850 | 0.009 | 89.927 | <0.001 |
| SOC08 | 0.976 | 0.010 | 93.333 | <0.001 |
| F2 BY |  |  |  |  |
| SOC05 | 0.907 | 0.005 | 170.184 | <0.001 |
| SOC06 | 0.908 | 0.006 | 152.360 | <0.001 |
| SOC07 | 0.882 | 0.007 | 124.032 | <0.001 |
| SOC09 | 0.917 | 0.007 | 135.191 | <0.001 |
| SOC10 | 0.889 | 0.007 | 134.941 | <0.001 |
| SOC11 | 0.888 | 0.007 | 126.160 | <0.001 |
| SOC16 | 0.933 | 0.006 | 154.580 | <0.001 |
| SOC26 | 0.718 | 0.015 | 48.051 | <0.001 |
| F3 BY |  |  |  |  |
| SOC12 | 0.907 | 0.005 | 175.932 | <0.001 |
| SOC13 | 0.915 | 0.005 | 180.317 | <0.001 |
| SOC14 | 0.885 | 0.005 | 163.629 | <0.001 |
| SOC15 | 0.896 | 0.006 | 144.986 | <0.001 |
| SOC17 | 0.908 | 0.005 | 166.786 | <0.001 |
| SOC22 | 0.863 | 0.007 | 115.502 | <0.001 |
| SOC23 | 0.881 | 0.007 | 125.050 | <0.001 |
| SOC24 | 0.861 | 0.007 | 124.304 | <0.001 |
| SOC25 | 0.801 | 0.011 | 74.325 | <0.001 |
| F4 BY |  |  |  |  |
| SOC18 | 0.915 | 0.006 | 151.193 | <0.001 |
| SOC19 | 0.954 | 0.003 | 333.243 | <0.001 |
| SOC20 | 0.953 | 0.003 | 298.469 | <0.001 |
| SOC21 | 0.942 | 0.007 | 140.365 | <0.001 |
| F2 WITH |  |  |  |  |
| F1 | 0.745 | 0.014 | 54.444 | <0.001 |
| F3 WITH |  |  |  |  |
| F1 | 0.749 | 0.013 | 59.557 | <0.001 |
| F2 | 0.905 | 0.006 | 157.368 | <0.001 |
| F4 WITH |  |  |  |  |
| F1 | 0.685 | 0.014 | 49.332 | <0.001 |
| F2 | 0.607 | 0.018 | 33.892 | <0.001 |
| F3 | 0.809 | 0.010 | 78.813 | <0.001 |
| Thresholds |  |  |  |  |
| SOC01$1 | -1.981 | 0.057 | -34.901 | <0.001 |
| SOC01$2 | -0.606 | 0.036 | -16.750 | <0.001 |
| SOC02$1 | -1.941 | 0.048 | -40.168 | <0.001 |
| SOC02$2 | -0.681 | 0.034 | -19.854 | <0.001 |
| SOC03$1 | -2.136 | 0.052 | -40.777 | <0.001 |
| SOC03$2 | -0.563 | 0.032 | -17.771 | <0.001 |
| SOC04$1 | -1.849 | 0.045 | -40.815 | <0.001 |
| SOC04$2 | -0.327 | 0.032 | -10.185 | <0.001 |
| SOC05$1 | -1.982 | 0.048 | -41.393 | <0.001 |
| SOC05$2 | -0.485 | 0.033 | -14.700 | <0.001 |
| SOC06$1 | -2.003 | 0.050 | -40.460 | <0.001 |
| SOC06$2 | -0.666 | 0.037 | -18.233 | <0.001 |
| SOC07$1 | -1.855 | 0.042 | -44.391 | <0.001 |
| SOC07$2 | -0.493 | 0.034 | -14.606 | <0.001 |
| SOC08$1 | -1.738 | 0.042 | -41.063 | <0.001 |
| SOC08$2 | -0.376 | 0.032 | -11.731 | <0.001 |
| SOC09$1 | -2.089 | 0.056 | -37.014 | <0.001 |
| SOC09$2 | -0.838 | 0.038 | -21.833 | <0.001 |
| SOC10$1 | -1.887 | 0.050 | -38.009 | <0.001 |
| SOC10$2 | -0.509 | 0.034 | -14.874 | <0.001 |
| SOC11$1 | -1.592 | 0.048 | -32.965 | <0.001 |
| SOC11$2 | -0.299 | 0.035 | -8.632 | <0.001 |
| SOC12$1 | -1.779 | 0.043 | -41.011 | <0.001 |
| SOC12$2 | -0.497 | 0.032 | -15.765 | <0.001 |
| SOC13$1 | -1.772 | 0.042 | -41.844 | <0.001 |
| SOC13$2 | -0.482 | 0.032 | -14.849 | <0.001 |
| SOC14$1 | -1.411 | 0.035 | -40.290 | <0.001 |
| SOC14$2 | -0.254 | 0.029 | -8.777 | <0.001 |
| SOC15$1 | -1.546 | 0.040 | -38.722 | <0.001 |
| SOC15$2 | -0.341 | 0.032 | -10.625 | <0.001 |
| SOC16$1 | -1.748 | 0.047 | -36.873 | <0.001 |
| SOC16$2 | -0.625 | 0.035 | -18.014 | <0.001 |
| SOC17$1 | -1.561 | 0.038 | -40.858 | <0.001 |
| SOC17$2 | -0.439 | 0.031 | -14.098 | <0.001 |
| SOC18$1 | -1.224 | 0.038 | -32.148 | <0.001 |
| SOC18$2 | 0.018 | 0.034 | 0.540 | 0.589 |
| SOC19$1 | -1.489 | 0.038 | -39.062 | <0.001 |
| SOC19$2 | -0.239 | 0.035 | -6.826 | <0.001 |
| SOC20$1 | -1.469 | 0.039 | -38.046 | <0.001 |
| SOC20$2 | -0.251 | 0.035 | -7.155 | <0.001 |
| SOC21$1 | -1.071 | 0.037 | -29.302 | <0.001 |
| SOC21$2 | 0.094 | 0.033 | 2.837 | 0.005 |
| SOC22$1 | -1.053 | 0.038 | -27.843 | <0.001 |
| SOC22$2 | 0.240 | 0.036 | 6.742 | <0.001 |
| SOC23$1 | -1.273 | 0.040 | -32.044 | <0.001 |
| SOC23$2 | -0.047 | 0.035 | -1.327 | 0.184 |
| SOC24$1 | -1.324 | 0.036 | -36.783 | <0.001 |
| SOC24$2 | -0.108 | 0.033 | -3.304 | 0.001 |
| SOC25$1 | -1.638 | 0.050 | -33.061 | <0.001 |
| SOC25$2 | -0.072 | 0.036 | -2.018 | 0.044 |
| SOC26$1 | -1.547 | 0.043 | -36.053 | <0.001 |
| SOC26$2 | -0.146 | 0.035 | -4.177 | <0.001 |
| *Emotional maturity* |  |  |  |  |
| F1 BY |  |  |  |  |
| EMOT01 | 0.880 | 0.006 | 139.861 | <0.001 |
| EMOT02 | 0.907 | 0.006 | 155.500 | <0.001 |
| EMOT03 | 0.938 | 0.004 | 245.913 | <0.001 |
| EMOT04 | 0.942 | 0.004 | 268.361 | <0.001 |
| EMOT05 | 0.907 | 0.005 | 180.794 | <0.001 |
| EMOT06 | 0.800 | 0.011 | 75.515 | <0.001 |
| EMOT07 | 0.833 | 0.009 | 88.911 | <0.001 |
| EMOT08 | 0.874 | 0.007 | 130.666 | <0.001 |
| F2 BY |  |  |  |  |
| EMOT09 | 0.430 | 0.029 | 14.617 | <0.001 |
| EMOT24 | 0.875 | 0.011 | 82.339 | <0.001 |
| EMOT25 | 0.972 | 0.004 | 239.733 | <0.001 |
| EMOT26 | 0.965 | 0.004 | 253.591 | <0.001 |
| EMOT27 | 0.872 | 0.012 | 73.331 | <0.001 |
| EMOT28 | 0.953 | 0.010 | 97.093 | <0.001 |
| EMOT29 | 0.726 | 0.018 | 41.149 | <0.001 |
| EMOT30 | 0.590 | 0.021 | 27.528 | <0.001 |
| F3 BY |  |  |  |  |
| EMOT10 | 0.902 | 0.005 | 167.834 | <0.001 |
| EMOT11 | 0.976 | 0.002 | 428.814 | <0.001 |
| EMOT12 | 0.958 | 0.004 | 273.313 | <0.001 |
| EMOT13 | 0.904 | 0.008 | 119.934 | <0.001 |
| EMOT14 | 0.887 | 0.008 | 106.902 | <0.001 |
| EMOT18 | 0.932 | 0.005 | 173.607 | <0.001 |
| EMOT19 | 0.930 | 0.007 | 137.925 | <0.001 |
| F4 BY |  |  |  |  |
| EMOT15 | 0.877 | 0.007 | 123.474 | <0.001 |
| EMOT16 | 0.909 | 0.006 | 159.371 | <0.001 |
| EMOT17 | 0.907 | 0.006 | 155.175 | <0.001 |
| EMOT20 | 0.928 | 0.006 | 152.617 | <0.001 |
| EMOT21 | 0.842 | 0.010 | 88.598 | <0.001 |
| EMOT22 | 0.852 | 0.008 | 107.742 | <0.001 |
| EMOT23 | 0.852 | 0.009 | 97.707 | <0.001 |
| F2 WITH |  |  |  |  |
| F1 | 0.159 | 0.031 | 5.122 | <0.001 |
| F3 WITH |  |  |  |  |
| F1 | -0.036 | 0.029 | -1.265 | 0.211 |
| F2 | 0.673 | 0.022 | 30.629 | <0.001 |
| F4 WITH |  |  |  |  |
| F1 | 0.152 | 0.029 | 5.336 | <0.001 |
| F2 | 0.711 | 0.018 | 39.965 | <0.001 |
| F3 | 0.905 | 0.006 | 142.828 | <0.001 |
| Thresholds |  |  |  |  |
| EMOT01$1 | -0.882 | 0.036 | -24.283 | <0.001 |
| EMOT01$2 | 0.373 | 0.034 | 10.850 | <0.001 |
| EMOT02$1 | -1.004 | 0.039 | -26.020 | <0.001 |
| EMOT02$2 | 0.151 | 0.035 | 4.333 | <0.001 |
| EMOT03$1 | -1.103 | 0.039 | -27.936 | <0.001 |
| EMOT03$2 | 0.160 | 0.034 | 4.703 | <0.001 |
| EMOT04$1 | -1.009 | 0.037 | -27.326 | <0.001 |
| EMOT04$2 | 0.169 | 0.034 | 4.970 | <0.001 |
| EMOT05$1 | -0.965 | 0.039 | -24.929 | <0.001 |
| EMOT05$2 | 0.308 | 0.035 | 8.861 | <0.001 |
| EMOT06$1 | -1.401 | 0.041 | -34.490 | <0.001 |
| EMOT06$2 | -0.150 | 0.035 | -4.286 | <0.001 |
| EMOT07$1 | -1.124 | 0.035 | -32.317 | <0.001 |
| EMOT07$2 | 0.167 | 0.035 | 4.813 | <0.001 |
| EMOT08$1 | -0.971 | 0.037 | -25.935 | <0.001 |
| EMOT08$2 | 0.294 | 0.036 | 8.165 | <0.001 |
| EMOT09$1 | -0.621 | 0.041 | -14.988 | <0.001 |
| EMOT09$2 | 0.093 | 0.040 | 2.298 | 0.022 |
| EMOT10$1 | -1.255 | 0.039 | -32.325 | <0.001 |
| EMOT10$2 | -0.462 | 0.035 | -13.227 | <0.001 |
| EMOT11$1 | -1.333 | 0.042 | -32.044 | <0.001 |
| EMOT11$2 | -0.640 | 0.039 | -16.506 | <0.001 |
| EMOT12$1 | -1.373 | 0.043 | -32.259 | <0.001 |
| EMOT12$2 | -0.683 | 0.039 | -17.402 | <0.001 |
| EMOT13$1 | -1.437 | 0.046 | -30.913 | <0.001 |
| EMOT13$2 | -0.790 | 0.041 | -19.362 | <0.001 |
| EMOT14$1 | -1.406 | 0.045 | -31.214 | <0.001 |
| EMOT14$2 | -0.547 | 0.038 | -14.277 | <0.001 |
| EMOT15$1 | -1.184 | 0.037 | -31.831 | <0.001 |
| EMOT15$2 | -0.248 | 0.033 | -7.528 | <0.001 |
| EMOT16$1 | -1.286 | 0.038 | -34.079 | <0.001 |
| EMOT16$2 | -0.289 | 0.034 | -8.544 | <0.001 |
| EMOT17$1 | -1.323 | 0.040 | -33.286 | <0.001 |
| EMOT17$2 | -0.248 | 0.034 | -7.257 | <0.001 |
| EMOT18$1 | -1.424 | 0.043 | -33.025 | <0.001 |
| EMOT18$2 | -0.545 | 0.036 | -15.199 | <0.001 |
| EMOT19$1 | -1.521 | 0.051 | -30.121 | <0.001 |
| EMOT19$2 | -0.750 | 0.042 | -17.996 | <0.001 |
| EMOT20$1 | -1.425 | 0.046 | -31.183 | <0.001 |
| EMOT20$2 | -0.532 | 0.037 | -14.569 | <0.001 |
| EMOT21$1 | -1.329 | 0.044 | -30.197 | <0.001 |
| EMOT21$2 | -0.217 | 0.035 | -6.295 | <0.001 |
| EMOT22$1 | -1.265 | 0.037 | -33.832 | <0.001 |
| EMOT22$2 | -0.154 | 0.033 | -4.669 | <0.001 |
| EMOT23$1 | -1.353 | 0.042 | -32.016 | <0.001 |
| EMOT23$2 | -0.210 | 0.031 | -6.694 | <0.001 |
| EMOT24$1 | -1.467 | 0.049 | -30.221 | <0.001 |
| EMOT24$2 | -0.543 | 0.038 | -14.417 | <0.001 |
| EMOT25$1 | -1.451 | 0.052 | -27.671 | <0.001 |
| EMOT25$2 | -0.533 | 0.038 | -14.134 | <0.001 |
| EMOT26$1 | -1.523 | 0.050 | -30.693 | <0.001 |
| EMOT26$2 | -0.559 | 0.038 | -14.800 | <0.001 |
| EMOT27$1 | -1.635 | 0.054 | -30.510 | <0.001 |
| EMOT27$2 | -0.895 | 0.043 | -20.819 | <0.001 |
| EMOT28$1 | -1.692 | 0.058 | -29.381 | <0.001 |
| EMOT28$2 | -0.847 | 0.044 | -19.324 | <0.001 |
| EMOT29$1 | -1.376 | 0.043 | -32.184 | <0.001 |
| EMOT29$2 | -0.276 | 0.034 | -8.076 | <0.001 |
| EMOT30$1 | -1.109 | 0.035 | -31.441 | <0.001 |
| EMOT30$2 | -0.097 | 0.036 | -2.714 | 0.007 |
| *Language and cognitive development* |  |  |  |  |
| F1 BY |  |  |  |  |
| LANCOG01 | 0.743 | 0.027 | 27.245 | <0.001 |
| LANCOG04 | 0.922 | 0.010 | 90.497 | <0.001 |
| LANCOG05 | 0.940 | 0.007 | 133.637 | <0.001 |
| LANCOG06 | 0.880 | 0.010 | 86.509 | <0.001 |
| LANCOG07 | 0.853 | 0.012 | 70.783 | <0.001 |
| LANCOG11 | 0.812 | 0.019 | 42.233 | <0.001 |
| LANCOG12 | 0.840 | 0.017 | 49.176 | <0.001 |
| LANCOG14 | 0.896 | 0.010 | 93.758 | <0.001 |
| F2 BY |  |  |  |  |
| LANCOG02 | 0.849 | 0.013 | 63.127 | <0.001 |
| LANCOG03 | 0.876 | 0.012 | 72.076 | <0.001 |
| LANCOG17 | 0.883 | 0.013 | 65.803 | <0.001 |
| LANCOG18 | 0.928 | 0.011 | 88.132 | <0.001 |
| LANCOG19 | 0.845 | 0.014 | 62.168 | <0.001 |
| F3 BY |  |  |  |  |
| LANCOG08 | 0.953 | 0.007 | 145.188 | <0.001 |
| LANCOG09 | 0.935 | 0.006 | 144.369 | <0.001 |
| LANCOG10 | 0.933 | 0.006 | 147.341 | <0.001 |
| LANCOG13 | 0.829 | 0.013 | 63.375 | <0.001 |
| LANCOG15 | 0.854 | 0.021 | 40.420 | <0.001 |
| LANCOG16 | 0.845 | 0.014 | 59.168 | <0.001 |
| F4 BY |  |  |  |  |
| LANCOG20 | 0.869 | 0.018 | 49.465 | <0.001 |
| LANCOG21 | 0.824 | 0.020 | 41.827 | <0.001 |
| LANCOG22 | 0.913 | 0.013 | 67.654 | <0.001 |
| LANCOG23 | 0.920 | 0.016 | 58.769 | <0.001 |
| LANCOG24 | 0.942 | 0.011 | 84.972 | <0.001 |
| LANCOG25 | 0.810 | 0.024 | 33.783 | <0.001 |
| LANCOG26 | 0.792 | 0.021 | 38.116 | <0.001 |
| F2 WITH |  |  |  |  |
| F1 | 0.821 | 0.012 | 67.430 | <0.001 |
| F3 WITH |  |  |  |  |
| F1 | 0.954 | 0.006 | 158.431 | <0.001 |
| F2 | 0.827 | 0.011 | 72.508 | <0.001 |
| F4 WITH |  |  |  |  |
| F1 | 0.878 | 0.012 | 71.021 | <0.001 |
| F2 | 0.770 | 0.018 | 42.188 | <0.001 |
| F3 | 0.804 | 0.017 | 46.385 | <0.001 |
| Thresholds |  |  |  |  |
| LANCOG01$1 | -1.816 | 0.048 | -37.756 | <0.001 |
| LANCOG02$1 | -1.007 | 0.034 | -29.650 | <0.001 |
| LANCOG03$1 | -0.514 | 0.033 | -15.511 | <0.001 |
| LANCOG04$1 | -1.462 | 0.044 | -33.471 | <0.001 |
| LANCOG05$1 | -1.237 | 0.040 | -30.755 | <0.001 |
| LANCOG06$1 | -0.764 | 0.035 | -22.034 | <0.001 |
| LANCOG07$1 | -0.800 | 0.037 | -21.430 | <0.001 |
| LANCOG08$1 | -1.053 | 0.039 | -26.665 | <0.001 |
| LANCOG09$1 | -0.629 | 0.038 | -16.551 | <0.001 |
| LANCOG10$1 | -0.580 | 0.038 | -15.245 | <0.001 |
| LANCOG11$1 | -1.281 | 0.041 | -31.284 | <0.001 |
| LANCOG12$1 | -1.375 | 0.042 | -32.704 | <0.001 |
| LANCOG13$1 | -0.512 | 0.031 | -16.527 | <0.001 |
| LANCOG14$1 | -0.961 | 0.037 | -25.800 | <0.001 |
| LANCOG15$1 | -1.654 | 0.055 | -29.860 | <0.001 |
| LANCOG16$1 | -0.619 | 0.039 | -16.008 | <0.001 |
| LANCOG17$1 | -0.981 | 0.035 | -28.144 | <0.001 |
| LANCOG18$1 | -0.711 | 0.033 | -21.709 | <0.001 |
| LANCOG19$1 | -0.779 | 0.035 | -22.153 | <0.001 |
| LANCOG20$1 | -1.554 | 0.043 | -36.052 | <0.001 |
| LANCOG21$1 | -1.121 | 0.043 | -26.178 | <0.001 |
| LANCOG22$1 | -1.591 | 0.048 | -33.002 | <0.001 |
| LANCOG23$1 | -1.730 | 0.053 | -32.758 | <0.001 |
| LANCOG24$1 | -1.245 | 0.043 | -28.875 | <0.001 |
| LANCOG25$1 | -1.501 | 0.052 | -28.595 | <0.001 |
| LANCOG26$1 | -1.281 | 0.046 | -28.078 | <0.001 |
| *Communication Skills and General Knowledge* |  |  |  |  |
| FIT BY |  |  |  |  |
| COMGEN01 | 0.887 | 0.007 | 134.538 | <0.001 |
| COMGEN02 | 0.862 | 0.008 | 101.959 | <0.001 |
| COMGEN03 | 0.944 | 0.004 | 230.791 | <0.001 |
| COMGEN04 | 0.925 | 0.005 | 175.521 | <0.001 |
| COMGEN05 | 0.908 | 0.006 | 154.946 | <0.001 |
| COMGEN06 | 0.919 | 0.005 | 176.483 | <0.001 |
| COMGEN07 | 0.878 | 0.007 | 122.449 | <0.001 |
| COMGEN08 | 0.666 | 0.017 | 38.854 | <0.001 |
| Thresholds |  |  |  |  |
| COMGEN01$1 | -1.572 | 0.037 | -42.033 | <0.001 |
| COMGEN01$2 | -0.375 | 0.031 | -12.283 | <0.001 |
| COMGEN02$1 | -1.618 | 0.040 | -40.650 | <0.001 |
| COMGEN02$2 | -0.435 | 0.030 | -14.403 | <0.001 |
| COMGEN03$1 | -1.022 | 0.033 | -31.328 | <0.001 |
| COMGEN03$2 | 0.083 | 0.032 | 2.581 | 0.010 |
| COMGEN04$1 | -0.757 | 0.036 | -20.798 | <0.001 |
| COMGEN04$2 | 0.276 | 0.036 | 7.760 | <0.001 |
| COMGEN05$1 | -1.383 | 0.040 | -34.164 | <0.001 |
| COMGEN05$2 | -0.291 | 0.034 | -8.584 | <0.001 |
| COMGEN06$1 | -1.378 | 0.040 | -34.642 | <0.001 |
| COMGEN06$2 | -0.311 | 0.032 | -9.671 | <0.001 |
| COMGEN07$1 | -1.386 | 0.036 | -38.495 | <0.001 |
| COMGEN07$2 | -0.427 | 0.033 | -13.120 | <0.001 |
| COMGEN08$1 | -1.554 | 0.048 | -32.486 | <0.001 |
| COMGEN08$2 | -0.323 | 0.038 | -8.573 | <0.001 |
