# Supplementary material for: Correction: Psychometric properties and factor structure of the Early Development Instrument in a sample of Jordanian children
Source: BMC Psychol. 2024 Feb 14;12:72. doi: 10.1186/s40359-023-01462-2 (PMC10865590; doi:10.1186/s40359-023-01462-2)
Supplement: Supplementary file 1 — Additional file 1. Corrected article body [file 40359_2023_1462_MOESM1_ESM.doc]

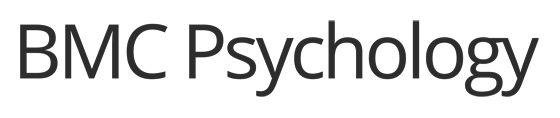
Ababneh *et al. BMC Psychology* 2022, **10**(1):316 https://doi.org/10.1186/s40359-022-01014-0

**RESEARCH ARTICLE**

**Open Access**

Psychometric properties and factor


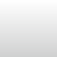

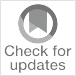


structure of the Early Development Instrument in a sample of Jordanian children

Emad G. Ababneh1,2,3, Eric K. Duku4* [,](http://orcid.org/0000-0003-3599-3527) Caroline Reid‑Westoby4, Ashley Gaskin4 and Magdalena Janus4

**Abstract**

**Background:** Investing in children’s early years can have a lasting positive effect, such as better academic outcomes throughout their school careers. In Jordan, investments have been made in early childhood development and early childhood care and education to improve children’s school readiness. School readiness comprises a range of abili‑ ties needed to succeed in school, including physical, emotional, social, and cognitive skills. To measure the impact of these investments on children’s school readiness, Jordan has been implementing the Early Development Instrument (EDI), a population‑level, teacher‑completed checklist of children’s school readiness, assessing children’s development in five main areas, referred to as domains.

**Methods:** The goal of the current study was to examine the psychometric properties of the Arabic version of the EDI, using data collected in 2018 on a sample of 5965 children in Jordan. The EDI was translated from the original English version to Arabic and adapted for use in Jordan. We conducted a categorical confirmatory factor analysis (CFA) for each of the five domains of the EDI and examined the reliability of the domains and subdomains using Cronbach’s alpha reliability coefficient.

**Results:** With few exceptions, the study results are in line with those of the analysis of the psychometric properties found with the original, Canadian English version of the EDI in a population of Canadian children. Results of CFAs demonstrated, for the most part, good model fits. Internal consistency indices of the domains ranged from 0.74 for physical health and well‑being to 0.96 for social competence. For the subdomains, they ranged from 0.42 to 0.94.

**Conclusions:** Our results provide empirical support for the adaptation of the EDI for population monitoring of school readiness in Jordan. Validation of the Arabic adaptation opens up the possibility of assessing school readiness of young children in Jordan in comparison to the many other countries that have successfully adapted and applied the EDI.

**Keywords:** Early development instrument (EDI), Jordan, Psychometric properties, Reliability, Validity

*Correspondence: [duku@mcmaster.ca](mailto:duku@mcmaster.ca)

4 Department of Psychiatry and Behavioural Neurosciences, Offord Centre for Child Studies, McMaster University, 293 Wellington St, North, Suite 132, Hamilton, ON L8L 8E7, Canada

Full list of author information is available at the end of the article

# Background

Investing in education from an early age has the poten- tial to have a long-term positive impact on children. The early years of life pave the way for children’s future devel- opment and success, both in school and in life [[1](#_bookmark6)]. Early childhood experiences affect the growth and develop- ment of the brain [[2](#_bookmark7)], creating neural connections that provide the basis for a range of skills, both cognitive and

© The Author(s) 2022. **Open Access** This article is licensed under a Creative Commons Attribution 4.0 International License, which permits use, sharing, adaptation, distribution and reproduction in any medium or format, as long as you give appropriate credit to the original author(s) and the source, provide a link to the Creative Commons licence, and indicate if changes were made. The images or other third party material in this article are included in the article’s Creative Commons licence, unless indicated otherwise in a credit line to the material. If material is not included in the article’s Creative Commons licence and your intended use is not permitted by statutory regulation or exceeds the permitted use, you will need to obtain permission directly from the copyright holder. To view a copy of this licence, visit <http://creativecommons.org/licenses/by/4.0/>. The Creative Commons Public Domain Dedication waiver ([http://creativeco](http://creativecommons.org/publicdomain/zero/1.0/) [mmons.org/publicdomain/zero/1.0/](http://creativecommons.org/publicdomain/zero/1.0/)) applies to the data made available in this article, unless otherwise stated in a credit line to the data.


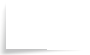

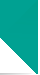

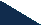


non-cognitive. It is therefore important to offer children a rich, stimulating environment to help them reach their full potential and be ready for school. School readiness is a holistic term that represents a child’s transition from their preschool to their school years and encompasses a range of abilities needed to succeed in school, such as physical, emotional, social, and cognitive skills [[3](#_bookmark8), [4](#_bookmark9)].

In Jordan, national efforts have been made to improve children’s school readiness and subsequent educational outcomes, including investments in early childhood development (ECD) and early childhood care and educa- tion (ECCE). In 2003, Jordan launched a program called the Education Reform for Knowledge Economy (ERfKE) which was developed to help students gain the neces- sary knowledge and skills to be competitive once they enter the workforce [[5](#_bookmark10)]. This program included improv- ing access to, and quality of, kindergarten classes [[6](#_bookmark11)], thus contributing to making this sector one of the most vital sectors in the educational system. In 2017, the Ministry of Education developed its strategic plan for the years 2018–2022, including a separate early education and early childhood development domain which related to the provision of quality programs for early education and childhood development in the second stage of kindergar- ten. This domain comprised two components: access and expansion, and quality, which included setting quality assurance criteria, and raising the proportion of kinder- garten children who are ready to learn based on the Early Development Instrument (EDI) from 76% in 2017 to 80%

in 2022 [[7](#_bookmark12)].

Jordan’s Modernization Vision for the year 2022 pos- tulated that education, especially early childhood educa- tion, is one of the engines of growth. The vision identified a set of initiatives to advance the sector so that by 2033 all children in Jordan would have an integrated, equita- ble system centered on the child to develop health care and education in early childhood [[8](#_bookmark13)]. Furthermore, the National Human Resources Development Strategy was developed for the years 2016–2025 to ensure that all chil- dren would have access to quality early childhood learn- ing and development experiences that promote primary school readiness, ensure healthy lives, and encourage their future well-being. In addition to that, the strategy aspired to ensure that children complete equitable and quality primary and secondary education, leading to rele- vant and effective learning outcomes. Moreover, it sought to increase the number of youth and adults who have relevant technical and vocational skills for employment, decent jobs, and entrepreneurship. In addition, the strat- egy works to guarantee fair access to affordable, relevant, and quality university education opportunities [[9](#_bookmark14)].

The Queen Rania Foundation conducted a study on the economic effects of investing in ECCE in Jordan.

The results of the study suggested that ECCE services for three years for Jordanian children would achieve benefits totaling $23,881 per child when the child finishes school and enters the labor market. The study showed that from a social point of view, the benefits of providing these ser- vices outweighed the costs, with a ratio of 1:9, meaning that every dollar invested in providing ECCE services would produce returns of $9 [[10](#_bookmark15)].

As with many regions in the Middle East and North Africa, about half of schools in Jordan are privately-run and therefore not accessible to a large segment of the population [[11](#_bookmark16)]. The ECCE policies developed as part of the ERfKE included establishing state nurseries and kindergartens. The focus on the first five years of life was dictated by existing and growing knowledge of how crucial the first five years of a child’s life are for optimal health and development [[12](#_bookmark17)].

In order to examine the impact of these policies on children’s school readiness, Jordan has turned to the EDI [[13](#_bookmark18)]. The EDI is a population-level, teacher-completed checklist covering five major developmental domains: physical health and well-being, social competence, emo- tional maturity, language and cognitive development, and communication skills and general knowledge. The EDI is a community-based instrument and data are typically aggregated to either the school, neighborhood, regional, or country level to provide a glimpse of how populations of children are doing. One advantage of the EDI is that it combines several domains of child development into one holistic measure which are all based on easily observable skills and behaviours [[13](#_bookmark18)]. The psychometric properties of the EDI have been examined in several countries [[13](#_bookmark18)– [22](#_bookmark25)], as well as with sub-populations of children [[23](#_bookmark26)–[25](#_bookmark27)]. The psychometric properties of the Arabic version of the EDI, implemented in Jordan, have yet to be examined.

# Current study

Measurement of children’s developmental status at school entry over time provides an opportunity to moni- tor investments in ECD and ECCE and examine their association with children’s concurrent and future out- comes. Thus, it is vital to establish a measurement that is psychometrically sound, reliable, and equitable. The goal of the current study was therefore to examine the psychometric characteristics of the Arabic version of the EDI using the data collected in 2018 on a sample of chil- dren in Jordan. Accordingly, this study sought to answer the following two research questions:

1. What are the psychometric properties (construct validity and internal consistency) of the five develop-

mental domains proposed by the developers in the Arabic version of the EDI?

1. Do the developmental domains of the Arabic ver- sion of the EDI have similar psychometric properties compared to the original English instrument?

# Methods

**Study design**

A cross-sectional study examining the validity and reli- ability of the Arabic version of the EDI in a sample of children attending first grade in Jordan in 2018 was conducted. The Ministry of Education and other educa- tional authorities were approached to obtain the schools’ approval to participate, and the study procedures included a pledge to the schools that the information to be collected is for scientific research purposes. SMS have been sent to parents about the study, not to obtain their consent, but to inform them of the study’s purposes only.

**Study sample**

The study sample came from a population of children enrolled in first grade during the 2017/2018 school year. The total number of children enrolled in first grade, based on data from the Educational Management Infor- mation System in the Ministry of Education, was 191,688, of which 98,570 (51.4%) were male. The study sample was selected in two stages. In the first stage, schools were selected to represent the location (i.e. rural or urban), the sex of children taught in the schools (males, females, mixed), the education directorate (Jordanian schools are affiliated with one of 43 education directorates, cover- ing the different regions of Jordan), and the geographical region (North, Middle, and South). As these characteris- tics were considered strata, the size of each stratum was determined according to its relative weight in the schools sampling frame. In the second stage, 24 children were selected from each of the chosen schools. In the event a school had less than 24 children, all children were selected to take part in the study. If, on the other hand, the number of children in a school was greater than 24, children were selected using a systematic random sample method. If the number of classes in a school was more than one, the children were distributed equally among the classes so that the required number was selected based on a systematic random sample. The final repre- sentative study sample comprised 6016 children from 260 schools. Sixty-four children were excluded because they had data missing on more than one domain of the EDI. The final analytic sample therefore comprised 5965 children with valid EDI data (99% of the original sample).

# Measures

**Early Development Instrument (EDI)**

The EDI [[13](#_bookmark18)] was developed in Canada to provide pop- ulation-level data on how children are doing in the year prior to their first year of formal schooling. The EDI is a 103-item, teacher-completed checklist used to assess children’s school readiness in five general areas of devel- opment: physical health and well-being, social com- petence, emotional maturity, language and cognitive development, and communication skills and general knowledge. These five domains are further broken down into 16 subdomains. Table [1](#_bookmark0) shows the domains and sub- domains of the EDI, as well as number of items in each domain. In addition to the 103 core items, the EDI con- tains questions about children’s demographic character- istics, their preschool experiences, the skills they possess, and the special problems they have (if any). It should be noted that these questions are not included in the com- putation of a child’s score on the five domains of the EDI. The EDI is completed by the teacher (or specialist in early childhood) in the second half of the school year, as this allows the respondent enough time to get to know the children in their class well and also allows children suf- ficient time to adapt to their new environment [[13](#_bookmark18)].

Domain scores are an average of the items in each domain and range from 0 to 10, with a higher score denoting greater ability. The mean scores are then divided into categories representing the highest and lowest scores in a given population. The distribution of scores is used to determine the percentage of children who are at differ- ent levels of school readiness. Children who score below the 10th percentile in a domain, based on a baseline or comparison population, are considered vulnerable in that area. The outcome measures used in this study were the scores on the five EDI domains.

The EDI has been well-validated as an assessment of child development (see [[26](#_bookmark28)] for a review). Several researchers have assessed and established the con- struct validity [[13](#_bookmark18), [17](#_bookmark21), [18](#_bookmark22)], predictive validity [[3](#_bookmark8), [27](#_bookmark29), [28](#_bookmark30)], between-group validity [[23](#_bookmark26), [29](#_bookmark31)], as well as cross-cultural validity [[15](#_bookmark19), [16](#_bookmark20), [19](#_bookmark23), [20](#_bookmark24)] of the EDI. The EDI is routinely collected at the population-level in Canada and Australia, and has been implemented in many countries, at vari- ous levels, including Brazil, Peru, China, Italy, Germany, Kyrgyzstan, Mexico, Jamaica, Indonesia, Vietnam, the United States, and the United Kingdom [[26](#_bookmark28)].

***Demographics and contextual characteristics***

In addition to the ratings of children’s development, other data on children, families and location were col- lected. Child’s sex at birth, age, maternal and paternal education were recorded on the EDI by teachers using

**Table 1** The five developmental domains of the EDI, the number of items in each domain, and the subdomains comprising each domain

**Domains Subdomains Example of items**

Physical health and well‑being (# items: 13) Physical readiness for school day Over or underdressed for school activities

Physical independence is independent in washroom habits

Gross and fine motor skills ability to manipulate objects

Social competence (# items: 26) Overall social competence Is able to play with various children;

Responsibility and respect follows rules and instructions

Approaches to learning listens attentively

Readiness to explore new things is eager to play with a new toy/game Emotional maturity (# items: 30) Prosocial and helpful behaviour Will try to help someone who has been hurt

Anxious and fearful behavior is upset when left by a parent/guardian

Aggressive behaviour gets into physical fights Hyperactive and inattentive behavior can’t sit still, is restless

Language and cognitive development (# items: 26)

Communication skills and general knowledge (# items: 8)

Basic literacy Is ale to attach sounds to letters

Interest in literacy/numeracy and memory is able to remember things easily Advanced literacy is able to read simple/complex words

Basic numeracy is able to count to 20

Communication skills and general knowledge Ability to tell a story; ability to take part in imagina‑

tive play

the child’s school-based information. Rurality and region were provided by the Educational Management Informa- tion System in the Ministry of Education. These variables are defined and described as follows:

*Child age* The child’s age at the time of EDI comple- tion was calculated in years. Child’s date of birth was obtained from the child’s profile at the school and documented by the child’s teacher on the EDI. Age was dichotomized as less than or equal to 6.65 years, and greater than 6.65 years.

*Child sex* The child’s sex was listed as either male or

female and was recorded by the child’s teacher on the first page of the EDI.

*School location (urban/rural)* Areas in Jordan are

divided into urban areas (localities with a popula- tion of 5,000 people or more) and rural areas (locali- ties with smaller populations). This information was provided by the Educational Management Informa- tion System in the Ministry of Education.

*Geographical area* In the year 2000, Jordan was

divided administratively into three regions: the North region, the Central region, and the South region. This information was provided by the Educa- tional Management Information System in the Min- istry of Education.

*Mother’s and father’s education* This variable repre-

sented the mother’s and father’s educational attain- ment levels, using information found in the child’s

school profile. The child’s teacher indicated this infor- mation on the first page of the EDI. Mother’s and father’s education were classified into of six levels: illiterate, less than general secondary school, general secondary school, community college diploma, bach- elor’s degree, and master’s degree or higher. These variables were then dichotomized as follows: lower education represented educational attainment up to and including general secondary school, and higher education, comprising community college diploma, bachelor’s degree, and master’s degree or higher.

**Jordan’s adaptation of the EDI**

In order to collect school readiness information in Jor- dan using the EDI, the instrument went through a rig- orous translation, adaptation, and verification process. The EDI was translated to Arabic by an early childhood expert, then it was assessed by university professors, researchers, and administrators in the childhood sector from the Ministry of Education, kindergarten supervi- sors working in the Ministry of Education and the private sector, and teachers in the field of early childhood. They judged the items in terms of the quality of the language and appropriateness for the purposes of the study. The views of these reviewers were taken in consideration, and a revised Arabic version of the EDI was produced and back-translated to English to verify that the significance of the items was not lost during the translation process.

The translation and feedback of the experts were then sent to the developers of the EDI in Canada for approval. The finalized Arabic version of the EDI was piloted on 1341 children. The internal consistency of the five EDI domains were estimated with Cronbach’s alpha, which ranged from 0.66 for the physical health and well-being domain to 0.93 for the social competence domain. This sample was also used to conduct an exploratory factor analysis (EFA) to assess the construct validity of the EDI, and the results revealed the same five domains as the original EDI factorial structure [[13](#_bookmark18)]. However, it should be noted that there was a significant difference on how items loaded on the factors [[30](#_bookmark32)]. A decision was made at the time to preserve the original factorial structure of the tool to assess the level of children’s readiness for school in Jordan, where the distribution of the EDI’s items on the domains remained the same as the original version. The EDI has been implemented three times in representative samples of children in Jordan so far: in 2010 as a baseline study, then in 2014 and 2018 as a follow-up study. Pre- liminary psychometric analyses were conducted on the 2010 sample (unpublished), however, the 2018 sample was chosen for this study as it comprised a much larger number of children and also better reflected the state of early childhood in Jordan, given all the changes that have occurred in the last decade.

**Statistical analysis plan**

The analysis for this study was conducted in three parts. First, we ran descriptive statistics on the background and demographic characteristics of the sample of chil- dren. Using the statistical software IBM SPSS, version 19 [[31](#_bookmark33)], we ran frequencies for categorical variables. Sec- ond, means and robust standard errors for domain scores were examined, overall and by sample demographic and contextual subgroups, accounting for clustering within schools, using the statistical software Stata, version 13.1 [[32](#_bookmark34)]. Third, we conducted a categorical confirmatory fac- tor analysis (CFA) for each of the four multidimensional domains and the one unidimensional domain of com- munication skills and general knowledge using Mplus

7.4 [[33](#_bookmark35)] to test the construct validity of the Arabic ver- sion of the EDI. Since the sampling methods employed meant that children were clustered within schools, we took clustering into account in our analysis. To evalu- ate model fit, we used multiple indices: the Comparative Fit Index (CFI), the Tucker–Lewis Index (TLI), the root mean square error of approximation (RMSEA), and the Weighted Root Mean Square Residual (WRMR). Another index of model fit computed was the chi-square (χ2) sta- tistic, which allows us to assess the adjustment between the model and the observed covariance matrix, with a lower value indicating a better adjustment, and with the

χ2 fit statistic ideally being non-significant [[34](#_bookmark36)]. Nev- ertheless, because the χ2 statistic is sensitive to sample size, we did not take this index into consideration in our analyses. We therefore based our model fit decisions on the CFI, TLI, RMSEA and WRMR indices. A value of

0.90 or greater is generally considered acceptable for the CFI and TLI, the RMSEA should be 0.06 or lower [[35](#_bookmark37)], and a recommended cut-off value of 1.0 for the WRMR is considered to indicate good fit [[36](#_bookmark38)]. We therefore based our model fit conclusions on these values. Last, we exam- ined the reliability (internal consistency) of each of the domains and subdomains using Cronbach’s alpha reliabil- ity coefficient in IBM SPSS, version 19 [[31](#_bookmark33)].

# Results

**Sample characteristics**

The study sample comprised 6016 children from across the country, of which 5965 (99.2%) had a valid EDI and were included in the analyses. Table [2](#_bookmark1) presents demo- graphic and contextual characteristics of children included in the final analytic sample. Just over half the sample was male (52.1%) and more than two thirds were enrolled in kindergarten (66.4%) prior to first grade. According to the data shown in Table [2](#_bookmark1), 47.6% of the chil- dren lived in rural areas. The table also shows that 39.4% of the children were from the Middle region of Jordan, while the percentages of children from the Northern and Southern regions were 30.4% and 21.3%, respectively.

**Mean scores by sample characteristics**

Next, we examined the scores on the EDI based on our sample characteristics, which are displayed in Table [3](#_bookmark2). Effect sizes and *p*-values corresponding to the differences in mean scores between the subgroups of the demo- graphic and contextual characteristics are presented in Additional file [1](#_bookmark5): Appendix A. EDI domain scores differed according to some demographic and contextual variables. For instance, females had higher scores than males on all EDI domains and the biggest mean differences were observed for the social competence and emotional matu- rity domains. Similarly, age was associated with scores on all of the domains and children older than the mean age of 6.65 years had higher scores on all EDI domains. Domain scores also differed based on both mother’s and father’s education, where the children whose parents had higher levels of education had higher scores. Here the big- gest mean differences were observed in three domains— social competence, language and cognitive development, and communication skills and general knowledge. Last, when examining scores on the EDI based on the location of the school, we observed higher domain scores for chil- dren attending schools in urban areas compared to those attending schools in rural regions.

**Table 2** Demographic and contextual characteristics of the final analytic sample

| **Variable** | **Category** | **Number of children** | **Percentage** |
| --- | --- | --- | --- |
| Child’s sex | Male | 3106 | 52.1% |
|  | Female | 2859 | 47.9% |
| Enrollment in kindergarten | Enrolled | 3958 | 66.4% |
|  | Not enrolled | 1697 | 28.4% |
| School location | Rural | 2836 | 47.6% |
|  | Urban | 2840 | 47.5% |
| Geographical area | South | 1272 | 21.3% |
|  | Middle | 2352 | 39.4% |
|  | North | 1816 | 30.4% |
| Mother education | Illiterate | 359 | 6.0% |
|  | Lower basic education (grades 1–6) | 542 | 9.1% |
|  | Higher basic education (grades 7–10) | 677 | 11.3% |
|  | Secondary education | 2093 | 35.1% |
|  | Diploma | 709 | 11.9% |
|  | University education | 1511 | 25.3% |
| Father education | Illiterate | 301 | 5.0% |
|  | Lower basic education (grades 1–6) | 731 | 12.3% |
|  | Higher basic education (grades 7–10) | 902 | 15.1% |
|  | Secondary education | 2356 | 39.5% |
|  | Diploma | 502 | 8.4% |
|  | University education | 1070 | 17.9% |
| Family monthly income | Less than 300JD ($423) | 2330 | 39.1% |
|  | 300JD‑599JD ($423–$845) | 2469 | 41.4% |
|  | 600JD‑899JD ($856–$1268) | 701 | 11.8% |
|  | More than 899JD ($1268) | 319 | 5.3% |

*Note.* Not all percentages add up to 100% because of missing values.

**Table 3** Means and robust standard errors of the developmental domains of the EDI by demographic and contextual characteristics, accounting for clustering within schools

| **Variables** | **Physical health and well-being** | **Social competence** | **Emotional maturity** | **Language and cognitive development** | **Communication skills and general knowledge** |
| --- | --- | --- | --- | --- | --- |
| Child’s sex |  |  |  |  |  |
| Female | 9.07 (0.04) | 8.13 (0.07) | 7.86 (0.07) | 8.63 (0.07) | 7.69 (0.09) |
| Male | 8.86 (0.05) | 7.63 (0.07) | 7.15 (0.07) | 8.33 (0.08) | 7.21 (0.08) |
| Child’s age |  |  |  |  |  |
| > 6.65 years | 8.99 (0.05) | 8.02 (0.07) | 7.54 (0.07) | 8.63 (0.07) | 7.64 (0.08) |
| ≤ 6.65 years | 8.93 (0.04) | 7.72 (0.06) | 7.45 (0.06) | 8.32 (0.07) | 7.23 (0.08) |
| Mother’s education |  |  |  |  |  |
| Higher education | 9.21 (0.04) | 8.37 (0.06) | 7.71 (0.07) | 9.08 (0.05) | 8.15 (0.07) |
| Lower education | 8.81 (0.05) | 7.57 (0.07) | 7.36 (0.07) | 8.12 (0.08) | 7.02 (0.09) |
| Father’s education |  |  |  |  |  |
| Higher education | 9.19 (0.05) | 8.43 (0.06) | 7.68 (0.09) | 9.12 (0.06) | 8.21 (0.08) |
| Lower education | 8.88 (0.05) | 7.68 (0.07) | 7.43 (0.06) | 8.26 (0.07) | 7.17 (0.08) |
| School location |  |  |  |  |  |
| Urban | 9.04 (0.05) | 8.11 (0.07) | 7.60 (0.08) | 8.68 (0.08) | 7.74 (0.09) |
| Rural | 8.90 (0.06) | 7.66 (0.09) | 7.40 (0.08) | 8.30 (0.08) | 7.17 (0.10) |

**Confirmatory factor analysis**

We ran a categorical CFA to examine the fit of each of

**Table 5** Cronbach’s alpha reliabilities for the five domains and 16 subdomains of the EDI

the five domains of the EDI. The results are summarized in Table [4](#_bookmark4) below (see Appendices B and C for results of the categorical CFAs using the 2010 and 2014 data, and

**Domains and subdomains Number of items**

**Reliability**

Additional file [1](#_bookmark5): Appendix D for detailed results show- ing thresholds and factor loadings). As can be seen in Table [4](#_bookmark4), the CFI and TLI for all five domains were higher than 0.92, indicating a good model fit. These indices were lowest for the emotional maturity domain, however. On the other hand, the RMSEA was higher than the criterion value 0.06 for two of the domains, that is, social compe- tence and communication skills and general knowledge. The WRMR was greater than 1 for all domains except physical health and well-being, indicating the fit was not as good for that domain.

**Internal consistency reliability**

The results indicated that, in this sample, the internal consistency reliability coefficient, estimated by Cron- bach’s alpha, for the five domains varied from 0.74 for physical health and well-being to 0.96 for the social competence domain. The subdomain reliabilities var- ied from 0.42 for physical independence to

0.94 for prosocial and helping behavior. Table [5](#_bookmark3) shows the internal consistencies for the various domains and subdomains.

# Discussion

The current study examined the psychometric proper- ties of the Arabic version of the EDI in a sample of 5952 children attending first grade in schools across Jordan. We found that EDI domain scores differed by children’s sex, socioeconomic characteristics, such as parental edu- cation and household income, and the location of the school. We found a consistent pattern between domains scores and age, similar to the findings with the original version of the EDI. Results of CFAs demonstrated, for the most part, good model fits. Internal consistency indices of the domains ranged from 0.74 for physical health and

Physical health and well‑being 13 0.74

Gross and fine motor skills 5 0.84

Physical readiness for school day 4 0.77

Physical independence 4 0.42

Social competence 26 0.96

Responsibility and respect 8 0.91

Approaches to learning 9 0.93

Overall social competence 5 0.86

Readiness to explore new things 4 0.91

Emotional maturity 30 0.93

Prosocial and helping behavior 8 0.94

Hyperactivity and inattention 7 0.92

Anxious and fearful behavior 8 0.85

Aggressive behavior 7 0.93

Language and cognitive development 26 0.93

Basic numeracy skills 7 0.82

Advanced literacy skills 6 0.83

Interest in literacy numeracy and memory 5 0.81

Basic literacy skills 8 0.84

Communication skills and general knowledge 8 0.92

well-being to 0.96 for social competence. For the subdo- mains, they ranged from 0.42 to 0.94.

Results of the CFA observed in our study are of similar magnitude to those of previous studies conducted else- where [[20](#_bookmark24), [37](#_bookmark39)]. For instance, the psychometric proper- ties of the EDI were examined in four countries (Canada, Australia, United States, and Jamaica) and the authors noted similar patterns of goodness of fit indices across the countries, with items tending to load on the same factor. Furthermore, in a sample of children in the Phil- ippines and Indonesia and using a shorter version of the EDI, Duku and colleagues [[37](#_bookmark39)] found adequate internal consistencies and parsimonious measurement models for all the domains, except physical health and well-being.

**Table 4** Results of the confirmatory factor analysis: goodness of fit statistics for the five EDI domains

|  | **χ2, df, *p* value** | **RMSEA** | **CFI** | **TLI** | **WRMR** |
| --- | --- | --- | --- | --- | --- |
| Physical health and well‑being | 370.070, 62, < 0.0001 | 0.029 (0.026, 0.032) | 0.979 | 0.974 | 1.674 |
| Social competence | 7688.092, 293, < 0.0001 | 0.065 (0.064, 0.066) | 0.933 | 0.925 | 4.435 |
| Emotional maturity | 6849.741, 399, < 0.0001 | 0.052 (0.051. 0.053) | 0.928 | 0.922 | 3.662 |
| Language and cognitive development | 1977.703, 293, < 0.0001 | 0.031 (0.030, 0.032) | 0.962 | 0.958 | 2.366 |
| Communication skills and general knowledge | 946.016, 20, < 0.0001 | 0.088 (0.083, 0.093) | 0.982 | 0.975 | 3.470 |

*Note*. RMSEA, root mean square error of approximation; CFI, comparative fit index; TLI, Tucker-Lewis index; WRMR, weighted root mean square residual; χ2, Chi-square test of fit; df, degrees of freedom

The authors noted the smaller number of items and the lack of variability in responses as possible reasons for the lower Cronbach’s alpha for the physical health and well- being domain. In contrast to these two studies, the physi- cal health and well-being domain had the best model fit of all the domains in the current study. Some potential explanations for these discrepancies include the fact the original structure of the EDI may not fully apply to the present study sample or that the structure of the instru- ment may have changed as a result of translation.

The internal consistency of the domains was generally very good (above 0.90), however, the physical health and well-being domain had a much lower Cronbach’s alpha. This domain in the original EDI had a Cronbach’s alpha of 0.84 [[13](#_bookmark18)], which is much higher than the one found in this sample (0.74). It has been suggested by others that this domain may not reflect a unidimensional construct [[38](#_bookmark40)], even though the original structure of the EDI con- sidered it as one. The authors argued that the subdomain of physical readiness for the school day may have less to do with the children and more to do with the behaviors of the children’s parents. Another possibility is that the subdomain of physical independence might be respon- sible, at least in part, for the poor reliability observed as the Cronbach’s alpha for this subdomain was 0.42. Pre- vious researchers have questioned the inclusion of the item of *sucks thumb/finger*, found within this subdomain, as a low factor loading and a weak correlation with the total score for the domain have been observed [[16](#_bookmark20), [38](#_bookmark40)]. It is important to note that the developers of the EDI men- tioned that seven items were kept even though they all had low factor loadings (less than 0.3) because teachers perceived these items to be important [[13](#_bookmark18)]. Three of these items were part of the physical health and well-being domain: independent in washroom habits, is well coor- dinated, and sucks a thumb/finger. More specifically, all three of these items comprise the physical independence subdomain which could explain why this subdomain had such a low Cronbach’s alpha.

Another potential explanation could be because of a smaller sample size compared to that used to examine the psychometric properties of the original EDI. Thus, a future study with a larger sample might be needed to confirm the internal consistency of the Arabic version of the EDI. One other potential reason for the lower Cron- bach’s alpha in this study could be the age of the children. The mean age in this study was 6.6 years whereas it was

5.6 years in the original study of the EDI [[13](#_bookmark18)]. The one- year difference in mean age may also explain the differ- ences in results observed in the current study compared to the original EDI study.

The pattern of internal consistency in Arabic EDI appears to be consistent with that seen in previous

research. All studies performed to date examining the internal consistency of the EDI, in various locations across the globe, have all found that the internal consist- ency was lowest for the physical health and well-being domain [[3](#_bookmark8), [13](#_bookmark18), [15](#_bookmark19), [19](#_bookmark23), [20](#_bookmark24), [39](#_bookmark41), [40](#_bookmark42)]. It is interesting to note that for studies examining the internal consistency of the EDI in a language other than English, the statistics tended to be lower [[16](#_bookmark20), [19](#_bookmark23), [37](#_bookmark39)].

**Strengths and limitations**

This study offers the first evidence of the reliability and validity of an Arabic adaptation of the internationally renowned EDI. Despite this strength, we acknowledge several limitations of our study. First, the data come from 2018 and therefore do not reflect the more recent contexts in which children are taught and educated, especially throughout the COVID-19 pandemic. Never- theless, data presented here might be used as a baseline if examining the impact of the pandemic on children’s development. Second, the results of the physical health and well-being domain suggest a weak reliability of this domain. As mentioned earlier, one reason behind this finding may be the translation of the instrument. In class- rooms taught by one teacher, it is difficult to examine inter-rater reliability, and we were not able to do so. We were also unable to assess test–retest reliability with this sample. Despite these limitations, the study strengths, such as its novelty, the large sample size, and potential for the use as a baseline in assessment on the impact of COVID-19, are considerable.

# Conclusion

This study evaluated the validity and reliability of the Arabic version of the EDI in a sample of children in Jor- dan. Our results provide empirical support for the adap- tation of the EDI for population monitoring of school readiness in this country. With few exceptions, our study results are in line with those of the analysis of the psycho- metric properties found with the original, English ver- sion of the EDI. Janus et al. [[20](#_bookmark24)] indicated that the items in each domain were put together conceptually, therefore we did not expect to have fit indices that met all the crite- ria for a good model fit. By validating the EDI in various countries, including Jordan, children’s school readiness can be monitored over time. Validation of the Arabic adaptation could also open up the possibility of compar- ing school readiness of young children in Jordan with the many other countries who have successfully adapted and applied the EDI.

**Abbreviations**

ECD: Early childhood development; ECCE: Early childhood care and education; ERfKE: Education reform for knowledge economy; EDI: Early Development

Instrument; EFA: Exploratory factor analysis; CFI: Comparative Fit Index; TLI: The Tucker–Lewis Index; RMSEA: Root mean square error of approximation; WRMR: Weighted root mean square residual; χ2: Chi‑square.

# Supplementary Information

The online version contains supplementary material available at [https://doi.](https://doi.org/10.1186/s40359-022-01014-0) [org/10.1186/s40359‑022‑01014‑0](https://doi.org/10.1186/s40359-022-01014-0).

**A****dditional file 1.** Supplementary material.

**Acknowledgements**

We thank those who contributed to the EDI survey: Jordan Ministry of Education, educational supervisors, teachers and parents, and UNICEF for its continued financial support.

**Author contributions**

EA, ED, and MJ contributed to the study conception and design. Material preparation, data collection, and statistical analysis were performed by EA and ED. The first draft of the manuscript was written by EA. CRW directed and managed all editing and revisions of the manuscript. EA, ED, CRW, AG and MJ

edited and commented on all versions of the manuscript. EA, ED, CRW, AG and MJ read and approved the final manuscript. All authors read and approved by the final manuscript.

**Funding**

The authors did not receive support from any organization for the submitted work.

**Availability of data and materials**

The datasets used and/or analysed during the current study are available from the corresponding author [ED] upon reasonable request.

**Declarations**

**Ethics approval and consent to participate**

The study received ethics approval from the National Center for Human Resources Development as well as from the UNICEF Jordan. All schools agreed to participate in the study, and data were collected through the children’s teacher and with the school’s approval.

**Consent for publication**

Not applicable.

**Competing interests**

The authors declare that they have no competing interests.

**Author details**

1National Center for Human Resources Development, Amman, Jordan. 2Jadara University, Irbid, Jordan. 3Amman Arab University, Amman, Jordan. 4Depart‑ ment of Psychiatry and Behavioural Neurosciences, Offord Centre for Child Studies, McMaster University, 293 Wellington St, North, Suite 132, Hamilton, ON L8L 8E7, Canada.

Received: 14 April 2022 Accepted: 3 December 2022

Published: 21 December 2022

**References**

1. Shonkoff JP, Phillips DA. From neurons to neighbourhoods: the science of early childhood development [Internet]. Washington, DC: National Acad‑ emies Press (US); 2000 [cited 2019 Jul 4]. 589 p. Available from: [https://](https://www.ncbi.nlm.nih.gov/books/NBK225558/)  [www.ncbi.nlm.nih.gov/books/NBK225558/.](https://www.ncbi.nlm.nih.gov/books/NBK225558/)
2. Hertzman C, Boyce T. How experience gets under the skin to cre‑ ate gradients in developmental health. Annu Rev Public Health. 2010;31(1):329–47.
3. Forget‑Dubois N, Lemelin JP, Boivin M, Dionne G, Séguin JR, Vitaro F, et al. Predicting early school achievement with the EDI: a longitudinal population‑based study. Early Educ Dev. 2007;18(3):405–26.
4. Janus M, Duku E. The school entry gap: socioeconomic, family, and health factors associated with children’s school readiness to learn. Early Educ Dev. 2007;18(3):375–403.
5. National Center for Human Resources Development (NCHRD). Completion Report (Education Reform for Knowledge Economy (ERfKEI):2003–2008. National Center for Human Resources Development; 2009.
6. Sultana RG. Jordan’s early childhood development initiative: making Jordan fit for children. UNICEF MENA‑RO. 2009;2:117.
7. Jordan Ministry of Education. Education Strategic Plan 2018–2022. Amman, Jordan; 2018. p. 158.
8. Hashemite Kingdom of Jordan. Economic Modernisation Vision [Internet]. Hashemite Kingdom of Jordan; 2022 [cited 2022 Aug 30]. p. 124. [https://](https://www.jordanvision.jo/en)  [www.jordanvision.jo/en.](https://www.jordanvision.jo/en)
9. National Committee for Human Resource Development. Education for Prosperity: Delivering Results—A National Strategy for Human Resource Development 2016–2025. Amman, Jordan; 2016. p. 276.
10. Queen Rania Foundation. Economic implications of investing in early childhood care and education in Jordan. 2017. p. 34.
11. Fink G, McCoy DC, Hatamleh HI, Pylvainen H, Chen A, Al‑Assaf G. Eco‑ nomic implications of investing in early childhood care & education in Jordan. Queen Rania Foundation; 2017. p. 33.
12. Children’s Bureau. Why The First 5 Years of Child Development Are So Important [Internet]. Child Abuse Prevention, Treatment & Welfare Services | Children’s Bureau. 2018 [cited 2021 Aug 25]. Available from: [https://www.all4kids.org/news/blog/why‑the‑first‑5‑years‑of‑child‑devel](https://www.all4kids.org/news/blog/why-the-first-5-years-of-child-development-are-so-important/)  [opment‑are‑so‑important/.](https://www.all4kids.org/news/blog/why-the-first-5-years-of-child-development-are-so-important/)
13. Janus M, Offord DR. Development and psychometric properties of the Early Development Instrument (EDI): a measure of children’s school readi‑ ness. Can J Behav Sci Revue Canadienne des Sciences du Comportement. 2007;39(1):1–22.
14. Brinkman SA, Silburn S, Lawrence D, Goldfeld S, Sayers M, Oberklaid F. Investigating the validity of the Australian Early Development Index. Early Educ Dev. 2007;18(3):427–51.
15. Brinkman SA, Kinnell A, Maika A, Hasan A, Jung H, Pradhan M. Validity and reliability of the Early Development Instrument in Indonesia. Child Indica‑ tors Res. 2017;10(2):331–52.
16. Georg S, Bosle C, Fischer JE, De Bock F. Psychometric properties and con‑ textual appropriateness of the German version of the Early Development Instrument. BMC Pediatrics. 2020;20(1):339.
17. Guhn M, Zumbo BD, Janus M, Hertzman C. Validation theory and research for a population‑level measure of children’s development, wellbeing, and school readiness. Soc Indicators Res. 2011;103(2):183–91.
18. Hymel S, LeMare L, McKee W. The Early Development Instrument: an examination of convergent and discriminant validity. Soc Indicators Res. 2011;103:267–82.
19. Ip P, Li SL, Rao N, Ng SSN, Lau WWS, Chow CB. Validation study of the Chinese Early Development Instrument (CEDI). BMC Pediatrics. 2013;13(1):146.
20. Janus M, Brinkman SA, Duku EK. Validity and psychometric properties of the Early Development Instrument in Canada, Australia, United States, and Jamaica. Soc Indicators Res. 2011;103(2):283.
21. Mousavi A, Krishnan V. Setting the scope for early child development instrument (EDI): a psychometric re‑examination of the tool with alberta data. Soc Indic Res. 2015;122(3):925–44.
22. Silburn SR, Brinkman S, Lawrence D, Sayers M, Goldfeld S, Oberklaid F. Establishing the construct and predictive validity of the Australian Early Development Index (AEDI). Early Hum Dev. 2007;1(83):S125.
23. Guhn M, Gadermann A, Zumbo BD. Does the EDI measure school readi‑ ness in the same way across different groups of children? Early Educ Dev. 2007;18(3):453–72.
24. Janus M, Zeraatkar D, Duku E, Bennett T. Validation of the Early Develop‑ ment Instrument for children with special health needs. J Paediatrics Child Health. 2018. <https://doi.org/10.1111/jpc.14264>.
25. Sam MA. An indigenous knowledges perspective on valid meaning mak‑ ing: a commentary on research with the EDI and aboriginal communities. Soc Indicator Res. 2011;103(2):315.
26. Janus M, Reid‑Westoby C. Monitoring the development of all children: the early development instrument. In: Moreno T, editor. Early childhood matters. The Hague: Bernard van Leer Foundation; 2016. p. 40–5.
27. Davies S, Janus M, Duku E, Gaskin A. Using the Early Development Instru‑ ment to examine cognitive and non‑cognitive school readiness and ele‑ mentary student achievement. Early Childhood Res Q. 2016;1(35):63–75.
28. Duncan RJ, Duncan GJ, Stanley L, Aguilar E, Halfon N. The kindergarten Early Development Instrument predicts third grade academic proficiency. Early Childhood Res Q. 2020;1(53):287–300.
29. Muhajarine N, Puchala C, Janus M. Does the EDI equivalently measure facets of school readiness for Aboriginal and non‑Aboriginal children? Soc Indicator Res. 2011;103(2):299–314.
30. Ababneh I, Hamed S. Early Development Instrument: a pilot study. National Center for Human Resource Development (NCHRD); 2008. p. 114.
31. IBM Corp. IBM SPSS Statistics for Windows, Version 19.0. Armonk: IBM Corp.; 2010.
32. StataCorp. Stata Statistical Software: Release 13. College Station: Stata‑ Corp LP; 2013.
33. Muthén LK, Muthén BO. Mplus user’s guide, 7th edn. Los Angeles: Muthén & Muthén; 1998. p. 876.
34. Little T. Longitudinal structural equation modeling. New York: The Guild‑ ford Press; 2013.
35. Hu L, Bentler PM. Cutoff criteria for fit indexes in covariance structure analysis: conventional criteria versus new alternatives. Struct Equ Model. 1999;6(1):1–55.
36. DiStefano C, Liu J, Jiang N, Shi D. Examination of the weighted root mean square residual: evidence for trustworthiness? Struct Equ Model. 2018;25(3):453–66.
37. Duku E, Janus M, Brinkman S. Investigation of the cross‑national equiva‑ lence of a measurement of early child development. Child Indicators Res. 2015;8(2):471–89.
38. Hagquist C, Hellström L. The psychometric properties of the early devel‑ opment instrument: a Rasch analysis based on Swedish pilot data. Soc Indicators Res. 2014;117(1):301–17.
39. Forer B, Zumbo BD. Validation of multilevel constructs: validation methods and empirical findings for the EDI. Soc Indicators Res. 2011;103(2):231.
40. Woolfson LM, Geddes R, McNicol S, Booth JN, Frank J. A cross‑sectional pilot study of the Scottish early development instrument: a tool for addressing inequality. BMC Public Health. 2013. [https://doi.org/10.1186/](https://doi.org/10.1186/1471-2458-13-1187) [1471‑2458‑13‑1187](https://doi.org/10.1186/1471-2458-13-1187).

# Publisher’s Note

Springer Nature remains neutral with regard to jurisdictional claims in pub‑ lished maps and institutional affiliations.

*Ready to submit your research ? Choose BMC and benefit from:*

- fast, convenient online submission
- thorough peer review by experienced researchers in your field
- rapid publication on acceptance
- support for research data, including large and complex data types
- gold Open Access which fosters wider collaboration and increased citations
- maximum visibility for your research: over 100M website views per year

**At BMC, research is always in progress. Learn more** biomedcentral.com/submissions
