# Supplementary material for: Psychometric properties and factor structure of the Early Development Instrument in a sample of Jordanian children
Source: BMC Psychol. 2022 Dec 21;10:316. doi: 10.1186/s40359-022-01014-0 (PMC9769466; doi:10.1186/s40359-022-01014-0)
Supplement: Supplementary file 1 [file 40359_2022_1014_MOESM1_ESM.docx]

**6. Appendices**

**6.1. Appendix A**

**Table A1**

***P-values from student’s t-tests and effect sizes (Cohen’s d) of the differences between subgroups for demographic and contextual characteristics for the developmental domains of the EDI and, accounting for clustering within schools***

| Variables | Physical Health & Well-being |  | Social Competence |  | Emotional Maturity |  | Language & Cognitive Development |  | Communication Skills & General Knowledge |  |
| --- | --- | --- | --- | --- | --- | --- | --- | --- | --- | --- |
|  | p-value | effect size | p-value | effect size | p-value | effect size | p-value | effect size | p-value | effect size |
| Child’s sex | 0.042 | -0.074 | <0.001 | -0.246 | <0.001 | -0.402 | <0.001 | -0.140 | <0.001 | -0.180 |
| Child’s age | 0.003 | -0.074 | <0.001 | -0.146 | 0.063 | -0.049 | <0.001 | -0.145 | <0.001 | -0.157 |
| Mother’s education | <0.001 | -0.268 | <0.001 | -0.409 | <0.001 | -0.192 | <0.001 | -0.487 | <0.001 | -0.453 |
| Father’s education | <0.001 | -0.261 | <0.001 | -0.392 | 0.001 | -0.137 | <0.001 | -0.451 | <0.001 | -0.429 |
| School location | 0.001 | -0.187 | <0.001 | -0.222 | 0.053 | -0.109 | 0.001 | -0.178 | <0.001 | -0.224 |

**6.2. Appendix B**

**Table B1**

***Results of the Confirmatory Factor Analysis: Goodness of fit statistics for the domains – 2010*** 

|  | χ^2^, df, p-value | RMSEA | CFI | TLI | WRMR |
| --- | --- | --- | --- | --- | --- |
| Physical Health & Well-being | 292.071, 62, <0.0001 | 0.030 (0.026, 0.033) | 0.982 | 0.977 | 1.567 |
| Social Competence | 5576.824, 293, <0.0001 | 0.065 (0.064, 0.067) | 0.952 | 0.947 | 3.761 |
| Emotional Maturity | 5226.729, 399, <0.0001 | 0.054 (0.052. 0.055) | 0.919 | 0.911 | 3.521 |
| Language and Cognitive Development | 1394.885, 293, <0.0001 | 0.031 (0.028, 0.031) | 0.972 | 0.969 | 2.045 |
| Communication Skills and General Knowledge | 642.514, 20, <0.0001 | 0.086 (0.080, 0.092) | 0.986 | 0.981 | 2.871 |

*Note.* RMSEA = Root mean square error of approximation, CFI = Comparative fit index, TLI = Tucker-Lewis index, WRMR = Weighted Root Mean Square Residual, χ^2^ = chi-square test of fit, df = degrees of freedom

**6.3. Appendix C**

**Table C1**

***Results of the Confirmatory Factor Analysis: Goodness of fit statistics for the five domains of the EDI*** 

|  | χ^2^, df, p-value | RMSEA | CFI | TLI | WRMR |
| --- | --- | --- | --- | --- | --- |
| Physical Health & Well-being | 380.800, 62, <0.0001 | 0.029 (0.027, 0.032) | 0.986 | 0.983 | 1.693 |
| Social Competence | 7629.134, 293, <0.0001 | 0.065 (0.064, 0.066) | 0.946 | 0.940 | 4.471 |
| Emotional Maturity | 7594.239, 399, <0.0001 | 0.055 (0.054. 0.056) | 0.916 | 0.908 | 4.866 |
| Language and Cognitive Development | 1748.280, 293, <0.0001 | 0.029 (0.028, 0.030) | 0.975 | 0.972 | 2.440 |
| Communication Skills and General Knowledge | 787.163, 20, <0.0001 | 0.080 (0.075, 0.085) | 0.988 | 0.984 | 3.462 |

*Note.* RMSEA = Root mean square error of approximation, CFI = Comparative fit index, TLI = Tucker-Lewis index, WRMR = Weighted Root Mean Square Residual, χ^2^ = chi-square test of fit, df = degrees of freedom

**6.4. Appendix D**

**Table D1**

***Results of the Categorical Confirmatory Factor Analysis 2018 – Standardized factor loadings and thresholds for the EDI domains***

| Domains | Estimate | S.E. | Est./S.E. | Two-Tailed P-Value |
| --- | --- | --- | --- | --- |
| *Physical Health and Well-being* |  |  |  |  |
| F1       BY |  |  |  |  |
| PHWB01 | 0.755 | 0.033 | 22.952 | <0.001 |
| PHWB02 | 0.903 | 0.02 | 46.156 | <0.001 |
| PHWB03 | 0.866 | 0.019 | 46.223 | <0.001 |
| PHWB04 | 0.853 | 0.024 | 35.861 | <0.001 |
| F2       BY |  |  |  |  |
| PHWB05 | 0.728 | 0.039 | 18.457 | <0.001 |
| PHWB06 | 0.47 | 0.04 | 11.798 | <0.001 |
| PHWB07 | 0.934 | 0.032 | 29.204 | <0.001 |
| PHWB13 | 0.385 | 0.057 | 6.813 | <0.001 |
| F3       BY |  |  |  |  |
| PHWB08 | 0.91 | 0.009 | 102.115 | <0.001 |
| PHWB09 | 0.959 | 0.006 | 170.741 | <0.001 |
| PHWB10 | 0.856 | 0.014 | 61.493 | <0.001 |
| PHWB11 | 0.807 | 0.012 | 65.857 | <0.001 |
| PHWB12 | 0.836 | 0.015 | 55.765 | <0.001 |
| F2 WITH |  |  |  |  |
| F1 | 0.311 | 0.05 | 6.221 | <0.001 |
| F3 WITH |  |  |  |  |
| F1 | 0.418 | 0.034 | 12.145 | <0.001 |
| F2 | 0.589 | 0.034 | 17.474 | <0.001 |
| Thresholds |  |  |  |  |
| PHWB01$1 | -1.192 | 0.051 | -23.539 | <0.001 |
| PHWB02$1 | -1.119 | 0.037 | -29.868 | <0.001 |
| PHWB03$1 | -1.189 | 0.038 | -31.601 | <0.001 |
| PHWB04$1 | -1.222 | 0.045 | -26.997 | <0.001 |
| PHWB05$1 | -1.616 | 0.054 | -29.993 | <0.001 |
| PHWB06$1 | -0.715 | 0.051 | -13.973 | <0.001 |
| PHWB07$1 | -1.388 | 0.042 | -33.266 | <0.001 |
| PHWB08$1 | -2.305 | 0.054 | -42.429 | <0.001 |
| PHWB08$2 | -1.156 | 0.039 | -29.667 | <0.001 |
| PHWB09$1 | -2.228 | 0.053 | -41.953 | <0.001 |
| PHWB09$2 | -0.973 | 0.038 | -25.707 | <0.001 |
| PHWB10$1 | -2.308 | 0.059 | -39.173 | <0.001 |
| PHWB10$2 | -1.22 | 0.045 | -27.099 | <0.001 |
| PHWB11$1 | -1.95 | 0.05 | -39.035 | <0.001 |
| PHWB11$2 | -0.719 | 0.034 | -21.214 | <0.001 |
| PHWB12$1 | -2.298 | 0.065 | -35.363 | <0.001 |
| PHWB12$2 | -1.119 | 0.038 | -29.312 | <0.001 |
| PHWB13$1 | -1.788 | 0.074 | -24.1 | <0.001 |
| PHWB13$2 | -1.23 | 0.059 | -20.949 | <0.001 |
| *Social competence* |  |  |  |  |
| F1       BY |  |  |  |  |
| SOC01 | 0.851 | 0.013 | 65.384 | <0.001 |
| SOC02 | 0.87 | 0.01 | 84.87 | <0.001 |
| SOC03 | 0.909 | 0.009 | 106.233 | <0.001 |
| SOC04 | 0.849 | 0.009 | 89.954 | <0.001 |
| SOC08 | 0.975 | 0.01 | 92.87 | <0.001 |
| F2       BY |  |  |  |  |
| SOC05 | 0.907 | 0.005 | 170.151 | <0.001 |
| SOC06 | 0.907 | 0.006 | 151.986 | <0.001 |
| SOC07 | 0.881 | 0.007 | 123.711 | <0.001 |
| SOC09 | 0.917 | 0.007 | 134.834 | <0.001 |
| SOC10 | 0.888 | 0.007 | 134.374 | <0.001 |
| SOC11 | 0.887 | 0.007 | 125.606 | <0.001 |
| SOC16 | 0.933 | 0.006 | 153.782 | <0.001 |
| SOC26 | 0.718 | 0.015 | 48.003 | <0.001 |
| F3       BY |  |  |  |  |
| SOC12 | 0.907 | 0.005 | 178.016 | <0.001 |
| SOC13 | 0.915 | 0.005 | 179.797 | <0.001 |
| SOC14 | 0.885 | 0.005 | 163.115 | <0.001 |
| SOC15 | 0.895 | 0.006 | 144.44 | <0.001 |
| SOC17 | 0.907 | 0.005 | 166.307 | <0.001 |
| SOC22 | 0.863 | 0.007 | 115.356 | <0.001 |
| SOC23 | 0.881 | 0.007 | 124.732 | <0.001 |
| SOC24 | 0.861 | 0.007 | 124.082 | <0.001 |
| SOC25 | 0.801 | 0.011 | 74.297 | <0.001 |
| F4       BY |  |  |  |  |
| SOC18 | 0.914 | 0.006 | 150.589 | <0.001 |
| SOC19 | 0.954 | 0.003 | 333.087 | <0.001 |
| SOC20 | 0.953 | 0.003 | 298.417 | <0.001 |
| SOC21 | 0.942 | 0.007 | 139.964 | <0.001 |
| F2       WITH |  |  |  |  |
| F1 | 0.745 | 0.014 | 54.253 | <0.001 |
| F3       WITH |  |  |  |  |
| F1 | 0.748 | 0.013 | 59.35 | <0.001 |
| F2 | 0.905 | 0.006 | 156.758 | <0.001 |
| F4       WITH |  |  |  |  |
| F1 | 0.684 | 0.014 | 49.121 | <0.001 |
| F2 | 0.606 | 0.018 | 33.84 | <0.001 |
| F3 | 0.808 | 0.01 | 78.755 | <0.001 |
| Thresholds |  |  |  |  |
| SOC01$1 | -1.983 | 0.057 | -34.82 | <0.001 |
| SOC01$2 | -0.605 | 0.036 | -16.694 | <0.001 |
| SOC02$1 | -1.943 | 0.048 | -40.109 | <0.001 |
| SOC02$2 | -0.681 | 0.034 | -19.821 | <0.001 |
| SOC03$1 | -2.135 | 0.052 | -40.765 | <0.001 |
| SOC03$2 | -0.563 | 0.032 | -17.804 | <0.001 |
| SOC04$1 | -1.85 | 0.045 | -40.752 | <0.001 |
| SOC04$2 | -0.328 | 0.032 | -10.215 | <0.001 |
| SOC05$1 | -1.984 | 0.048 | -41.347 | <0.001 |
| SOC05$2 | -0.486 | 0.033 | -14.744 | <0.001 |
| SOC06$1 | -2.006 | 0.05 | -40.409 | <0.001 |
| SOC06$2 | -0.667 | 0.036 | -18.29 | <0.001 |
| SOC07$1 | -1.856 | 0.042 | -44.35 | <0.001 |
| SOC07$2 | -0.494 | 0.034 | -14.649 | <0.001 |
| SOC08$1 | -1.739 | 0.042 | -40.997 | <0.001 |
| SOC08$2 | -0.377 | 0.032 | -11.768 | <0.001 |
| SOC09$1 | -2.092 | 0.057 | -36.946 | <0.001 |
| SOC09$2 | -0.84 | 0.038 | -21.917 | <0.001 |
| SOC10$1 | -1.888 | 0.05 | -37.933 | <0.001 |
| SOC10$2 | -0.51 | 0.034 | -14.925 | <0.001 |
| SOC11$1 | -1.592 | 0.048 | -32.906 | <0.001 |
| SOC11$2 | -0.3 | 0.035 | -8.653 | <0.001 |
| SOC12$1 | -1.782 | 0.044 | -40.82 | <0.001 |
| SOC12$2 | -0.498 | 0.031 | -15.812 | <0.001 |
| SOC13$1 | -1.773 | 0.042 | -41.793 | <0.001 |
| SOC13$2 | -0.483 | 0.032 | -14.897 | <0.001 |
| SOC14$1 | -1.411 | 0.035 | -40.292 | <0.001 |
| SOC14$2 | -0.255 | 0.029 | -8.791 | <0.001 |
| SOC15$1 | -1.546 | 0.04 | -38.718 | <0.001 |
| SOC15$2 | -0.342 | 0.032 | -10.65 | <0.001 |
| SOC16$1 | -1.749 | 0.048 | -36.809 | <0.001 |
| SOC16$2 | -0.626 | 0.035 | -18.075 | <0.001 |
| SOC17$1 | -1.561 | 0.038 | -40.797 | <0.001 |
| SOC17$2 | -0.439 | 0.031 | -14.133 | <0.001 |
| SOC18$1 | -1.224 | 0.038 | -32.134 | <0.001 |
| SOC18$2 | 0.019 | 0.034 | 0.549 | 0.583 |
| SOC19$1 | -1.489 | 0.038 | -39.063 | <0.001 |
| SOC19$2 | -0.239 | 0.035 | -6.827 | <0.001 |
| SOC20$1 | -1.469 | 0.039 | -38.042 | <0.001 |
| SOC20$2 | -0.251 | 0.035 | -7.163 | <0.001 |
| SOC21$1 | -1.071 | 0.037 | -29.289 | <0.001 |
| SOC21$2 | 0.095 | 0.033 | 2.867 | 0.004 |
| SOC22$1 | -1.052 | 0.038 | -27.819 | <0.001 |
| SOC22$2 | 0.24 | 0.035 | 6.768 | <0.001 |
| SOC23$1 | -1.272 | 0.04 | -32.021 | <0.001 |
| SOC23$2 | -0.047 | 0.035 | -1.325 | 0.185 |
| SOC24$1 | -1.324 | 0.036 | -36.769 | <0.001 |
| SOC24$2 | -0.108 | 0.033 | -3.286 | 0.001 |
| SOC25$1 | -1.639 | 0.05 | -32.98 | <0.001 |
| SOC25$2 | -0.073 | 0.036 | -2.051 | 0.04 |
| SOC26$1 | -1.547 | 0.043 | -35.987 | <0.001 |
| SOC26$2 | -0.146 | 0.035 | -4.183 | <0.001 |
| *Emotional maturity* |  |  |  |  |
| F1       BY |  |  |  |  |
| EMOT01 | 0.88 | 0.006 | 139.806 | <0.001 |
| EMOT02 | 0.907 | 0.006 | 155.366 | <0.001 |
| EMOT03 | 0.938 | 0.004 | 245.386 | <0.001 |
| EMOT04 | 0.942 | 0.004 | 268.208 | <0.001 |
| EMOT05 | 0.908 | 0.005 | 184.094 | <0.001 |
| EMOT06 | 0.8 | 0.011 | 75.483 | <0.001 |
| EMOT07 | 0.832 | 0.009 | 88.818 | <0.001 |
| EMOT08 | 0.875 | 0.007 | 130.639 | <0.001 |
| F2       BY |  |  |  |  |
| EMOT09 | 0.43 | 0.029 | 14.628 | <0.001 |
| EMOT24 | 0.875 | 0.011 | 82.211 | <0.001 |
| EMOT25 | 0.971 | 0.004 | 238.848 | <0.001 |
| EMOT26 | 0.964 | 0.004 | 252.924 | <0.001 |
| EMOT27 | 0.872 | 0.012 | 72.996 | <0.001 |
| EMOT28 | 0.953 | 0.01 | 96.794 | <0.001 |
| EMOT29 | 0.726 | 0.018 | 41.085 | <0.001 |
| EMOT30 | 0.59 | 0.021 | 27.489 | <0.001 |
| F3       BY |  |  |  |  |
| EMOT10 | 0.902 | 0.005 | 167.586 | <0.001 |
| EMOT11 | 0.976 | 0.002 | 428.691 | <0.001 |
| EMOT12 | 0.958 | 0.004 | 273.316 | <0.001 |
| EMOT13 | 0.904 | 0.008 | 119.878 | <0.001 |
| EMOT14 | 0.887 | 0.008 | 106.819 | <0.001 |
| EMOT18 | 0.932 | 0.005 | 173.382 | <0.001 |
| EMOT19 | 0.93 | 0.007 | 137.791 | <0.001 |
| F4       BY |  |  |  |  |
| EMOT15 | 0.877 | 0.007 | 123.288 | <0.001 |
| EMOT16 | 0.909 | 0.006 | 159.253 | <0.001 |
| EMOT17 | 0.907 | 0.006 | 155.094 | <0.001 |
| EMOT20 | 0.928 | 0.006 | 152.49 | <0.001 |
| EMOT21 | 0.842 | 0.01 | 88.538 | <0.001 |
| EMOT22 | 0.852 | 0.008 | 107.608 | <0.001 |
| EMOT23 | 0.852 | 0.009 | 97.681 | <0.001 |
| F2       WITH |  |  |  |  |
| F1 | 0.159 | 0.031 | 5.106 | <0.001 |
| F3       WITH |  |  |  |  |
| F1 | -0.036 | 0.029 | -1.251 | 0.211 |
| F2 | 0.674 | 0.022 | 30.642 | <0.001 |
| F4       WITH |  |  |  |  |
| F1 | 0.153 | 0.029 | 5.336 | <0.001 |
| F2 | 0.711 | 0.018 | 39.965 | <0.001 |
| F3 | 0.905 | 0.006 | 142.828 | <0.001 |
| Thresholds |  |  |  |  |
| EMOT01$1 | -0.881 | 0.036 | -24.255 | <0.001 |
| EMOT01$2 | 0.372 | 0.034 | 10.809 | <0.001 |
| EMOT02$1 | -1.003 | 0.039 | -25.98 | <0.001 |
| EMOT02$2 | 0.151 | 0.035 | 4.334 | <0.001 |
| EMOT03$1 | -1.103 | 0.04 | -27.899 | <0.001 |
| EMOT03$2 | 0.161 | 0.034 | 4.707 | <0.001 |
| EMOT04$1 | -1.008 | 0.037 | -27.292 | <0.001 |
| EMOT04$2 | 0.169 | 0.034 | 4.976 | <0.001 |
| EMOT05$1 | -0.967 | 0.039 | -24.928 | <0.001 |
| EMOT05$2 | 0.307 | 0.035 | 8.862 | <0.001 |
| EMOT06$1 | -1.401 | 0.041 | -34.427 | <0.001 |
| EMOT06$2 | -0.15 | 0.035 | -4.259 | <0.001 |
| EMOT07$1 | -1.125 | 0.035 | -32.254 | <0.001 |
| EMOT07$2 | 0.167 | 0.035 | 4.797 | <0.001 |
| EMOT08$1 | -0.972 | 0.038 | -25.872 | <0.001 |
| EMOT08$2 | 0.293 | 0.036 | 8.125 | <0.001 |
| EMOT09$1 | -0.62 | 0.041 | -14.954 | <0.001 |
| EMOT09$2 | 0.093 | 0.041 | 2.292 | 0.022 |
| EMOT10$1 | -1.256 | 0.039 | -32.268 | <0.001 |
| EMOT10$2 | -0.462 | 0.035 | -13.198 | <0.001 |
| EMOT11$1 | -1.333 | 0.042 | -32.013 | <0.001 |
| EMOT11$2 | -0.639 | 0.039 | -16.475 | <0.001 |
| EMOT12$1 | -1.373 | 0.043 | -32.241 | <0.001 |
| EMOT12$2 | -0.683 | 0.039 | -17.382 | <0.001 |
| EMOT13$1 | -1.437 | 0.046 | -30.896 | <0.001 |
| EMOT13$2 | -0.79 | 0.041 | -19.331 | <0.001 |
| EMOT14$1 | -1.406 | 0.045 | -31.194 | <0.001 |
| EMOT14$2 | -0.546 | 0.038 | -14.253 | <0.001 |
| EMOT15$1 | -1.184 | 0.037 | -31.813 | <0.001 |
| EMOT15$2 | -0.247 | 0.033 | -7.496 | <0.001 |
| EMOT16$1 | -1.286 | 0.038 | -34.184 | <0.001 |
| EMOT16$2 | -0.289 | 0.034 | -8.545 | <0.001 |
| EMOT17$1 | -1.323 | 0.04 | -33.343 | <0.001 |
| EMOT17$2 | -0.248 | 0.034 | -7.269 | <0.001 |
| EMOT18$1 | -1.423 | 0.043 | -33.004 | <0.001 |
| EMOT18$2 | -0.545 | 0.036 | -15.203 | <0.001 |
| EMOT19$1 | -1.521 | 0.051 | -30.102 | <0.001 |
| EMOT19$2 | -0.75 | 0.042 | -17.999 | <0.001 |
| EMOT20$1 | -1.425 | 0.046 | -31.172 | <0.001 |
| EMOT20$2 | -0.532 | 0.037 | -14.568 | <0.001 |
| EMOT21$1 | -1.329 | 0.044 | -30.187 | <0.001 |
| EMOT21$2 | -0.217 | 0.035 | -6.286 | <0.001 |
| EMOT22$1 | -1.266 | 0.037 | -33.784 | <0.001 |
| EMOT22$2 | -0.153 | 0.033 | -4.667 | <0.001 |
| EMOT23$1 | -1.352 | 0.042 | -31.995 | <0.001 |
| EMOT23$2 | -0.21 | 0.031 | -6.672 | <0.001 |
| EMOT24$1 | -1.466 | 0.049 | -30.205 | <0.001 |
| EMOT24$2 | -0.544 | 0.038 | -14.418 | <0.001 |
| EMOT25$1 | -1.452 | 0.053 | -27.597 | <0.001 |
| EMOT25$2 | -0.533 | 0.038 | -14.147 | <0.001 |
| EMOT26$1 | -1.524 | 0.05 | -30.624 | <0.001 |
| EMOT26$2 | -0.559 | 0.038 | -14.817 | <0.001 |
| EMOT27$1 | -1.636 | 0.054 | -30.454 | <0.001 |
| EMOT27$2 | -0.896 | 0.043 | -20.876 | <0.001 |
| EMOT28$1 | -1.691 | 0.058 | -29.343 | <0.001 |
| EMOT28$2 | -0.848 | 0.044 | -19.363 | <0.001 |
| EMOT29$1 | -1.376 | 0.043 | -32.128 | <0.001 |
| EMOT29$2 | -0.276 | 0.034 | -8.076 | <0.001 |
| EMOT30$1 | -1.109 | 0.035 | -31.361 | <0.001 |
| EMOT30$2 | -0.096 | 0.036 | -2.694 | 0.007 |
| *Language and cognitive development* |  |  |  |  |
| F1       BY |  |  |  |  |
| LANCOG01 | 0.743 | 0.027 | 27.198 | <0.001 |
| LANCOG04 | 0.922 | 0.01 | 90.172 | <0.001 |
| LANCOG05 | 0.939 | 0.007 | 132.951 | <0.001 |
| LANCOG06 | 0.879 | 0.01 | 86.208 | <0.001 |
| LANCOG07 | 0.852 | 0.012 | 70.342 | <0.001 |
| LANCOG11 | 0.812 | 0.019 | 42.182 | <0.001 |
| LANCOG12 | 0.84 | 0.017 | 49.105 | <0.001 |
| LANCOG14 | 0.895 | 0.01 | 93.135 | <0.001 |
| F2       BY |  |  |  |  |
| LANCOG02 | 0.849 | 0.013 | 63 | <0.001 |
| LANCOG03 | 0.876 | 0.012 | 71.89 | <0.001 |
| LANCOG17 | 0.883 | 0.013 | 65.79 | <0.001 |
| LANCOG18 | 0.928 | 0.011 | 88.129 | <0.001 |
| LANCOG19 | 0.845 | 0.014 | 62.148 | <0.001 |
| F3       BY |  |  |  |  |
| LANCOG08 | 0.953 | 0.007 | 144.495 | <0.001 |
| LANCOG09 | 0.934 | 0.006 | 143.805 | <0.001 |
| LANCOG10 | 0.933 | 0.006 | 146.789 | <0.001 |
| LANCOG13 | 0.829 | 0.013 | 63.232 | <0.001 |
| LANCOG15 | 0.854 | 0.021 | 40.237 | <0.001 |
| LANCOG16 | 0.846 | 0.014 | 59.123 | <0.001 |
| F4       BY |  |  |  |  |
| LANCOG20 | 0.869 | 0.018 | 49.364 | <0.001 |
| LANCOG21 | 0.824 | 0.02 | 41.735 | <0.001 |
| LANCOG22 | 0.913 | 0.013 | 67.745 | <0.001 |
| LANCOG23 | 0.921 | 0.016 | 58.491 | <0.001 |
| LANCOG24 | 0.942 | 0.011 | 84.956 | <0.001 |
| LANCOG25 | 0.81 | 0.024 | 33.74 | <0.001 |
| LANCOG26 | 0.792 | 0.021 | 38.003 | <0.001 |
| F2       WITH |  |  |  |  |
| F1 | 0.823 | 0.012 | 67.676 | <0.001 |
| F3       WITH |  |  |  |  |
| F1 | 0.954 | 0.006 | 157.836 | <0.001 |
| F2 | 0.827 | 0.011 | 72.722 | <0.001 |
| F4       WITH |  |  |  |  |
| F1 | 0.88 | 0.012 | 72.726 | <0.001 |
| F2 | 0.771 | 0.018 | 42.801 | <0.001 |
| F3 | 0.806 | 0.017 | 47.144 | <0.001 |
| Thresholds |  |  |  |  |
| LANCOG01$1 | -1.817 | 0.048 | -37.757 | <0.001 |
| LANCOG02$1 | -1.007 | 0.034 | -29.631 | <0.001 |
| LANCOG03$1 | -0.513 | 0.033 | -15.48 | <0.001 |
| LANCOG04$1 | -1.463 | 0.044 | -33.456 | <0.001 |
| LANCOG05$1 | -1.238 | 0.04 | -30.781 | <0.001 |
| LANCOG06$1 | -0.763 | 0.035 | -22.014 | <0.001 |
| LANCOG07$1 | -0.8 | 0.037 | -21.403 | <0.001 |
| LANCOG08$1 | -1.054 | 0.039 | -26.693 | <0.001 |
| LANCOG09$1 | -0.629 | 0.038 | -16.545 | <0.001 |
| LANCOG10$1 | -0.58 | 0.038 | -15.241 | <0.001 |
| LANCOG11$1 | -1.283 | 0.041 | -31.213 | <0.001 |
| LANCOG12$1 | -1.375 | 0.042 | -32.642 | <0.001 |
| LANCOG13$1 | -0.511 | 0.031 | -16.477 | <0.001 |
| LANCOG14$1 | -0.961 | 0.037 | -25.767 | <0.001 |
| LANCOG15$1 | -1.655 | 0.055 | -29.813 | <0.001 |
| LANCOG16$1 | -0.618 | 0.039 | -15.979 | <0.001 |
| LANCOG17$1 | -0.98 | 0.035 | -28.122 | <0.001 |
| LANCOG18$1 | -0.71 | 0.033 | -21.675 | <0.001 |
| LANCOG19$1 | -0.779 | 0.035 | -22.111 | <0.001 |
| LANCOG20$1 | -1.552 | 0.043 | -36.01 | <0.001 |
| LANCOG21$1 | -1.121 | 0.043 | -26.132 | <0.001 |
| LANCOG22$1 | -1.592 | 0.048 | -32.977 | <0.001 |
| LANCOG23$1 | -1.732 | 0.053 | -32.64 | <0.001 |
| LANCOG24$1 | -1.244 | 0.043 | -28.849 | <0.001 |
| LANCOG25$1 | -1.501 | 0.053 | -28.566 | <0.001 |
| LANCOG26$1 | -1.282 | 0.046 | -28.025 | <0.001 |
| *Communication Skills and General Knowledge* |  |  |  |  |
| FIT      BY |  |  |  |  |
| COMGEN01 | 0.886 | 0.007 | 134.004 | <0.001 |
| COMGEN02 | 0.861 | 0.008 | 101.515 | <0.001 |
| COMGEN03 | 0.944 | 0.004 | 230.488 | <0.001 |
| COMGEN04 | 0.925 | 0.005 | 175.136 | <0.001 |
| COMGEN05 | 0.907 | 0.006 | 154.298 | <0.001 |
| COMGEN06 | 0.919 | 0.005 | 175.762 | <0.001 |
| COMGEN07 | 0.878 | 0.007 | 121.999 | <0.001 |
| COMGEN08 | 0.667 | 0.017 | 38.844 | <0.001 |
| Thresholds |  |  |  |  |
| COMGEN01$1 | -1.572 | 0.038 | -41.916 | <0.001 |
| COMGEN01$2 | -0.374 | 0.031 | -12.229 | <0.001 |
| COMGEN02$1 | -1.619 | 0.04 | -40.535 | <0.001 |
| COMGEN02$2 | -0.434 | 0.03 | -14.363 | <0.001 |
| COMGEN03$1 | -1.021 | 0.033 | -31.233 | <0.001 |
| COMGEN03$2 | 0.085 | 0.032 | 2.651 | 0.008 |
| COMGEN04$1 | -0.756 | 0.036 | -20.733 | <0.001 |
| COMGEN04$2 | 0.278 | 0.035 | 7.831 | <0.001 |
| COMGEN05$1 | -1.383 | 0.041 | -34.041 | <0.001 |
| COMGEN05$2 | -0.29 | 0.034 | -8.558 | <0.001 |
| COMGEN06$1 | -1.378 | 0.04 | -34.521 | <0.001 |
| COMGEN06$2 | -0.31 | 0.032 | -9.644 | <0.001 |
| COMGEN07$1 | -1.386 | 0.036 | -38.372 | <0.001 |
| COMGEN07$2 | -0.426 | 0.033 | -13.09 | <0.001 |
| COMGEN08$1 | -1.555 | 0.048 | -32.413 | <0.001 |
| COMGEN08$2 | -0.325 | 0.038 | -8.61 | <0.001 |
